# Capturing Ultrafast Spin Dynamics in Single-Molecule Magnets Using Femtosecond X-ray Emission Spectroscopy

**DOI:** 10.1021/acs.jpclett.5c00383

**Published:** 2025-04-17

**Authors:** Kyle Barlow, Ryan Phelps, Julien Eng, Rebecca A. Ingle, Dmitry Khakhulin, Mykola Biednov, Sharmistha Paul Dutta, Yifeng Jiang, Frederico A. Lima, Vandana Tiwari, Christopher Milne, Tetsuo Katayama, Marco Coletta, Euan K. Brechin, Thomas J. Penfold, J. Olof Johansson

**Affiliations:** † EaStCHEM School of Chemistry, University of Edinburgh, David Brewster Road, Edinburgh, EH9 3FJ, U.K.; ‡ Chemistry, School of Natural and Environmental Sciences, Newcastle University, NE1 7RU, Newcastle upon Tyne, U.K.; § Department of Chemistry, University College London, 20 Gordon Street, London, WC1H 0AJ, U.K.; ∥ European XFEL GmbH, Holzkoppel 4, 22869 Schenefeld, Germany; ⊥ Japan Synchrotron Radiation Research Institute, Kouto 1-1-1, Sayo, Hyogo 679-5198, Japan; # RIKEN SPring-8 Center, 1-1-1 Kouto, Sayo, Hyogo 679-5148, Japan

## Abstract

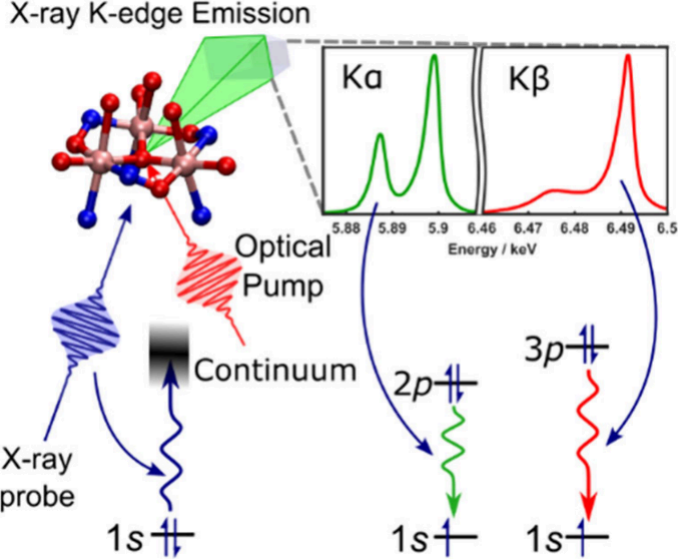

Achieving ultrafast photomagnetic switching of single-molecule magnets (SMMs) could lead to simultaneous fast and dense data storage devices. To facilitate this, a thorough understanding of the ultrafast dynamics emerging after ultrashort laser pulse excitation is essential. However, the complex nature of these materials means there is a lack of established experimental techniques that can probe the spin dynamics in SMMs. Herein, we perform femtosecond time-resolved Mn K-edge X-ray emission spectroscopy on a Mn(III)-based trinuclear SMM (Mn_3_) and the model system Mn(acac)_3_. The spectral changes of Mn(acac)_3_ are consistent with switching between Jahn–Teller distorted structures expected after photoexcitation. A similar result is observed for Mn_3_; however, the Kβ signal also reveals insight into the distribution of spin states populated within 100 fs. The importance of using probes across the electromagnetic spectrum to gain a thorough understanding of the dynamics of exchange-coupled complexes is highlighted.

Magnetic materials are used to store data where the direction of the magnetization dictates the state of the data bit (one or zero). Currently, small electromagnets are used to reorient the direction of the magnetic moments of the material to write the data. Unfortunately, this method introduces an upper bound of how quickly data can be written,^1^ which is becoming problematic considering the dramatic increased demand for data storage technologies. In the past few decades, femtosecond laser pulses have been used to manipulate the magnetization of a material faster than ever before.^2−5^ One state-of-the-art method is to use light to control the magnetocrystalline anisotropy.^6−8^ Magnetocrystalline anisotropy arises from spin–orbit coupling and the crystal-field environment to provide a preferential direction for the magnetization. Using light to manipulate the crystal field can lead to a photoinduced anisotropy, which applies a torque to the magnetic moments to change the direction of the magnetization.^9,10^

Despite the many successes, only a few condensed phase materials have the desired ground and excited state properties that can lead to efficient photomagnetic switching. To overcome this, it is instructive to investigate the huge number of molecule-based magnets that have been developed in recent years.^11−14^ They are interesting because they tend to have much greater synthetic flexibility, which could be exploited to provide the optimum properties for photomagnetic switching.^15−20^ Of these, single-molecule magnets (SMMs) provide additional advantages including their nanometer size that increases data storage density.^21−26^ Manganese(III)-based SMMs were among the first SMMs developed.^21^ Octahedral, high-spin *d*^4^ manganese(III) ions display Jahn–Teller distortion due to degeneracy in the *e*_g_ (*d*_*z*^2^_ and *d*_*x*^2^–*y*^2^_) orbitals.^27^ The nature of this distortion dictates the magnetocrystalline anisotropy.^28^ If the distorted structure is axially elongated (when the *d*_*z*^2^_ orbital is populated), then the lower energy state is when the spin magnetic moments have the maximum *z*-axis component (*M*_*S*_ = ±*S*). This is known as easy axis anisotropy. Alternatively, when the *d*_*x*^2^–*y*^2^_ orbital is populated, an axially compressed geometry is obtained, and the magnetic moments preferentially point in the *xy*-plane (*M*_*S*_ = 0), known as easy plane anisotropy. Therefore, by photoexciting an electron between the two *e*_g_ orbitals, it is possible to switch the Jahn–Teller distortion and change the anisotropy from easy axis to easy plane or vice versa.^29^

Our previous femtosecond optical and X-ray experiments investigating Mn(III) complexes and SMMs have provided significant new insight into the nuclear motion and time scales involved in the excited state dynamics.^29−34^ Upon photoexcitation of the crystal-field transitions, these complexes display vibrational wavepackets^30,31^ directly related to changes in the Jahn–Teller distortion and consequently changes in magnetic anisotropy. In ref,^30^ two Mn(III) complexes were studied, namely, Mn(acac)_3_ where acac = acetylacetonate and [Mn_3_O(Et-sao)_3_(β-pic)_3_(ClO_4_)], where saoH_2_ and β-pic are salicylaldoxime and β-picoline (3-methylpyridine),^35^ respectively, now referred to as Mn_3_. Mn_3_ has three Mn(III) ions with *s* = 2 ferromagnetically coupled via the superexchange interaction to give a total spin of *S* = 6.^35^ Optical^30^ and X-ray K-edge^34^ transient absorption spectroscopies show the activation of coherent Jahn–Teller modes after crystal-field excitation. In Mn(acac)_3_, this leads to a long-lived (>400 ps) axially compressed species. However, the rigidity of Mn_3_ restricts any significant nuclear motion with bond lengths changing by a maximum of 0.05 Å and reduces the excited state lifetime to less than 10 ps.^34^ Despite the detailed insight on the nuclear dynamics, these spectroscopies do not have the spin sensitivity required to directly inform on the magnetization dynamics.

Time-resolved K-edge X-ray emission spectroscopy (TR-XES) has previously been shown to carry significantly more information on spin states than optical or X-ray absorption. In particular, the Kβ emission from molecules depends heavily on the 3*d*-3*p* exchange energy and is therefore sensitive to valence spin structure.^36^ This has been used successfully to track the spin states after photon absorption in mononuclear Fe(II)^37−39^ and other complexes.^40,41^ In these cases, there exists only a small number of spin states that are accessed, and the time-resolved difference spectra can be compared to a set of reference compounds. However, for large polynuclear exchange-coupled transition metal complexes, such as Mn_3_, there are many spin states and no suitable reference spectra. Therefore, it is interesting to explore the dynamics of these complex systems using X-ray emission to unravel what information this technique holds.

The Kα and Kβ emission spectra of Mn(acac)_3_ and Mn_3_ in ethanol (EtOH) are shown in Figure 1. The Kα spectra (Figure 1a) are composed of a lower energy 2*p*_1/2_ to 1*s* (Kα_2_) transition and higher energy 2*p*_3/2_ to 1*s* transition (Kα_1_), which occur after core ionization of the 1*s* electrons. The positions of the two peaks are sensitive to the charge density on the metal ions, and the splitting of the peaks depends upon the strength of the Mn atomic 2*p* spin–orbit coupling. The asymmetry in the transition lineshapes arises from multiplet effects.^42^ The Kβ spectra (Figure 1b) are composed of 3*p* to 1*s* transitions. The shape and splitting of these peaks (low-energy Kβ’ and high-energy Kβ_1,3_) are also sensitive to the charge density on the metal but, due to the stronger 3*d*-3*p* exchange interaction (compared to the 3*d*-2*p* exchange interaction involved in Kα), exhibit increased sensitivity to the valence spin structure.^43,44^

**Figure 1:**
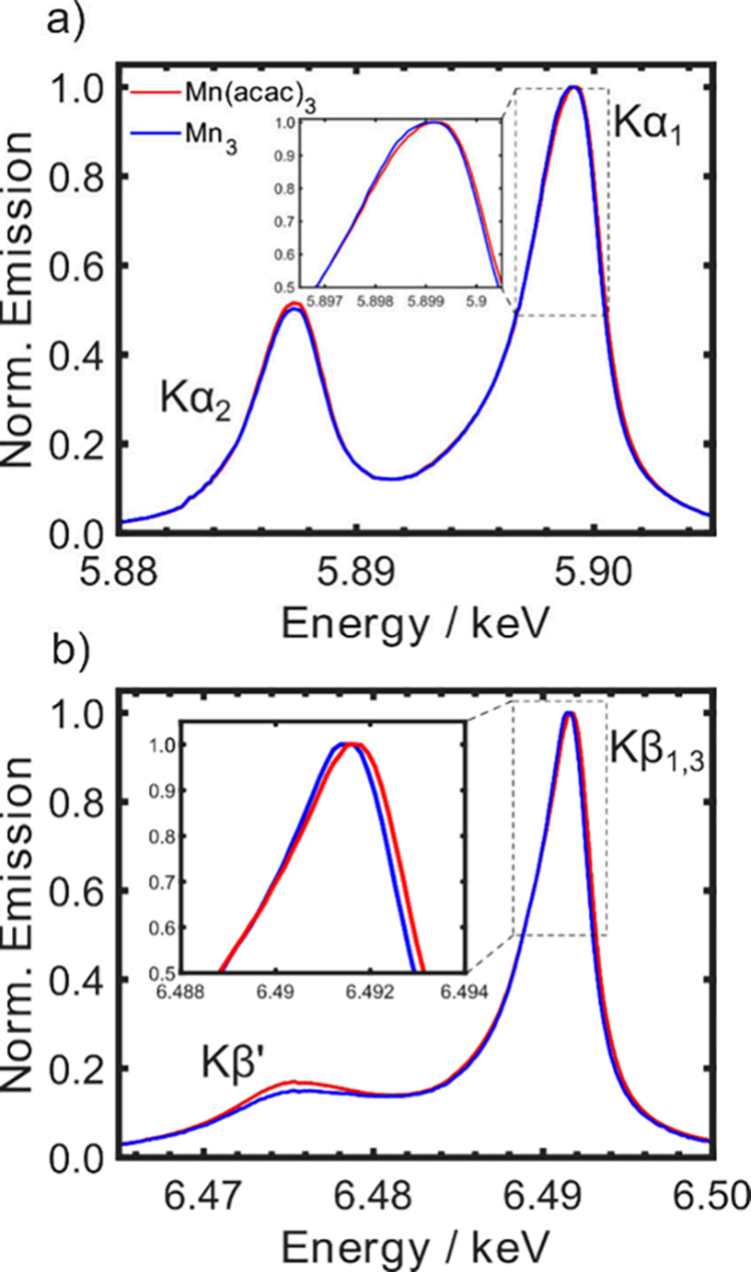
Normalized ground state K-edge emission spectra of Mn(acac)_3_ (red) and Mn_3_ (green) in EtOH, showing the a) Kα spectra and b) Kβ spectra for the two complexes. The insets in a) and b) focus on the Kα_1_ and Kβ_1,3_ peaks, respectively.

The Kα spectra for the two complexes are very similar, which is expected, as they both are composed of Mn ions in the same +3 oxidation state. A slightly larger splitting (0.3 eV) between the Kβ peaks and a more intense Kβ’ peak is observed for Mn(acac)_3_ than Mn_3_. Since each Mn(III) ion formally has *s* = 2, the slightly lower Kβ’ peak intensity and reduced splitting for Mn_3_ suggests that the spin density on the metal ions is lower than Mn(acac)_3_. This observation could be caused by two effects. First, the superexchange interactions lead to ferromagnetic coupling between the metal centers in Mn_3_, and consequently a portion of the spin density is found on the ligands rather than the Mn ions, i.e., a higher metal–ligand covalency. Second, spin–orbit coupling may reduce the effective spin state of a molecule by coupling together states of lower spin multiplicity into the ground state. In terms of spin states of Mn(acac)_3_ and Mn_3_, Tables S1 and S2 show the low-lying excited spin states, calculated using the NEVPT2 level of theory (see Supporting Information for details). Indeed, Mn(acac)_3_ has a quintet ground state that is well-separated (>1 eV) from all the other states. On the other hand, Mn_3_ has a significant number of lower-spin states that lie within 2 meV of the electronic ground state, which can therefore change the effective spin state due to spin–orbit coupling and thermal population. The high density of different spin states close to the ground state is in good agreement with magnetic susceptibility measurements that suggest the first excited states are only a few meV above the *S* = 6 ground state.^35^

The TR-XES difference spectra after photoexcitation using a 400 nm pump to excite the same metal-centered transition in both complexes^30^ (Figure S2) are shown in Figure 2 for various time delays. The Kα spectra (Figures 2a and 2c) for Mn(acac)_3_ and Mn_3_ exhibit the same derivative-like shape that is indicative of a blue shift in the spectrum. This is consistent with an increase in electronic charge density and spin state on the metal ions. However, as Kα is typically less sensitive to spin state due to the weaker 3*d*-2*p* exchange interaction and the spin state of Mn(acac)_3_ is expected to exhibit little change during the dynamics, especially at longer times, we ascribe this to the change in charge density in a manner consistent with the observations of ref.^45^ Indeed, the *d*-orbitals that are populated via photoexcitation have a weaker mixing with the ligands. The Jahn–Teller distortion initiated by the photoexcitation increases the equatorial metal–ligand bonds from 1.93 to 2.07 Å as shown by CASSCF//NEVPT2 calculations.^30^ This transfers electron density toward the metal ions and decreases their effective nuclear charge and shifts the emission to higher energy. This is supported by NEVPT2 simulations of the Kα emission spectra in Figure 3a and 3c, which shows the ground state (axial Jahn–Teller distortion) and two transient Kα XES for Mn(acac)_3_ in its quintet state. The first corresponds to the difference between the equatorial Jahn–Teller distorted structure and the ground state, while the second corresponds to a constrained Jahn–Teller distortion aimed at mimicking the smaller structural change around the Mn sites in Mn_3_. The direct simulation of the latter (i.e., Mn_3_) is too computationally expensive due to the size of the active space and the number of states involved. The structures are based on the results from a previous X-ray absorption paper where the nuclear geometry in the relaxed excited state was determined.^34^ Both of the transient spectra exhibit the same expected blue shift observed experimentally, consistent with previous X-ray K-edge absorption data of Mn_3_ where the spectral changes of the pre-edge suggest a weaker interaction between the metal ions and the ligands after photoexcitation.^34^

**Figure 2:**
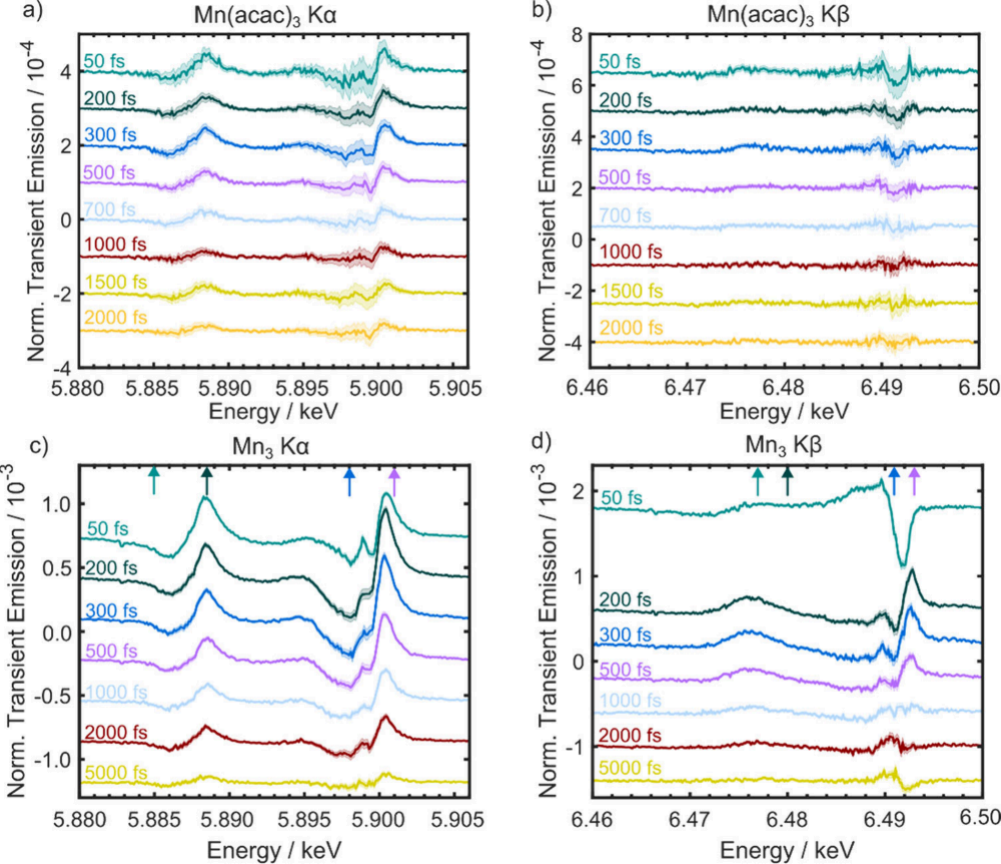
Transient difference emission spectra at different time delays after 400 nm photoexcitation. These have been normalized with respect to the ground state spectrum. The shaded areas describe the standard error of the mean (66% confidence interval). They have been offset on the *y*-axis to aid visualization. a) Kα spectra of Mn(acac)_3_. b) Kβ spectra of Mn(acac)_3_. c) Kα spectra of Mn_3_. d) Kβ spectra of Mn_3_. The arrows at the top of panels c) and d) indicate the energies of the probe plotted in Figure 4.

**Figure 3:**
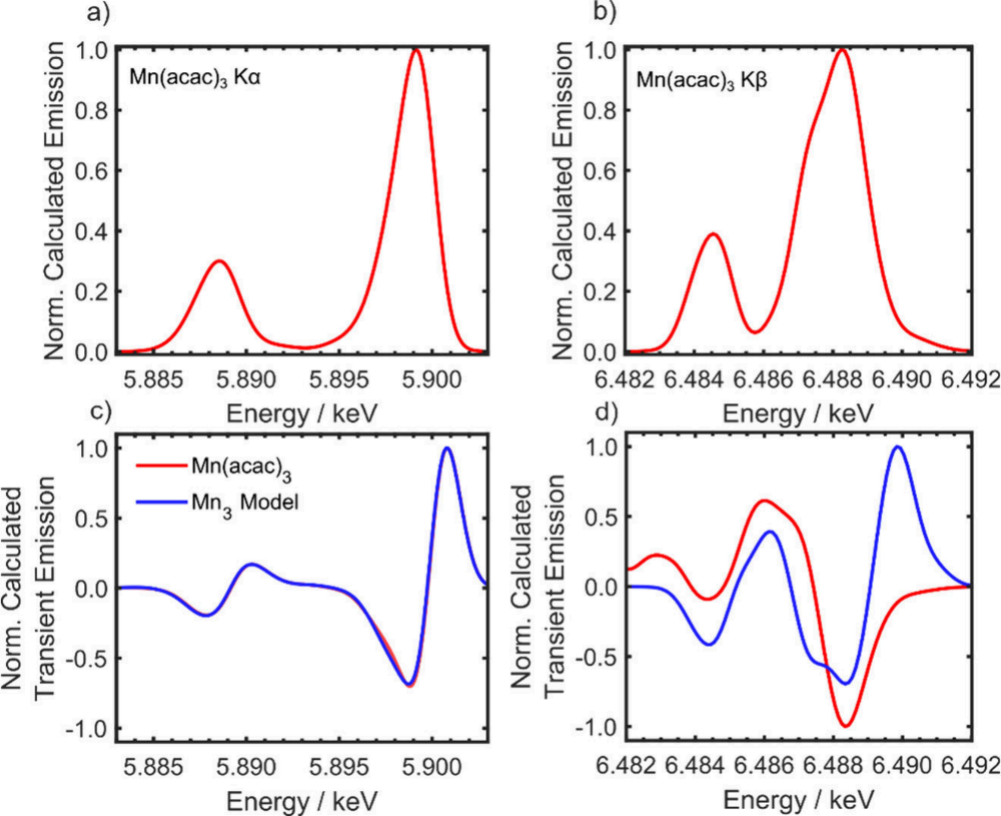
Calculated X-ray emission spectra of Mn(acac)_3_ in the ground and excited state. a) Calculated Kα emission spectrum of Mn(acac)_3_. b) Calculated Kβ emission spectrum of Mn(acac)_3_. (c,d) Calculated transient X-ray emission spectra. The spectra of Mn(acac)_3_ have been calculated using the optimized structure in the first excited state. The spectra of Mn_3_ have been simulated using Mn(acac)_3_ and mimicking the known small structural distortions of the coordination sphere of Mn_3_ in the excited state.^34^

The Kβ difference spectra after 400 nm excitation are shown in Figures 2b and 2d. Similar to the Kα spectra, the Kβ spectra can inform on the effective nuclear charge on the metal ions. However, more importantly for the current study, these spectra exhibit a larger dependence on the spin state and spin density due to the 3*d*-3*p* exchange interactions, which are more significant than observed for Kα spectra due to wave function overlap. Following the dynamics in Mn(acac)_3_ is challenging due to the low signal-to-noise ratio in the measurements. This is due to the absorption cross section at the pump wavelength for Mn(acac)_3_ being around 10 times lower than the absorption cross section at 400 nm for Mn_3_.^30^ Nevertheless, the transient spectra appear to exhibit little change during the dynamics and are dominated by a red shift of the Kβ_1,3_ peak, which is most apparent between 300 and 700 fs. Figure 3d shows the calculated NEVPT2 Kβ transient spectrum for the change to equatorial Jahn–Teller elongation, which exhibits the red shift observed experimentally (e.g., = 500 fs, Figure 2b).

The Kβ transient spectra of Mn_3_ exhibit a much higher signal-to-noise ratio, which allows us to extract more information on the spin dynamics. At early times (50 fs), the Kβ_1,3_ band exhibits a red shift compared with the ground state. However, within 200 fs, the transient band blue shifts, comparable to the shifts observed in the Kα spectra. At 5 ps, the band returned to a value lower than the ground state value (i.e., a red shift). Given that the Kα spectra only show a blue shift of the emission bands, the complex time evolution of the Kβ emission cannot be explained by changes in effective nuclear charge alone. Changes in the complex’s spin structure, reflected in the spectra through the 3*d*-3*p* exchange interactions, must be responsible for the changes in the Kβ emission.^42,46^Figure 3d shows the calculated NEVPT2 Kβ ground state and transient spectrum for the constrained Jahn–Teller distortion that mimics the small structural change around the Mn sites in Mn_3_,^34^ and this exhibits the blue shift observed between 200–1000 fs. The red shift occurring at early and late times can be described in terms of the spin state in the absence of structural change. At 50 fs, there is little time for structural changes to occur, whereas at later times (>4 ps) the complex has largely returned to the ground state. Indeed, the K-edge pre-edge absorption measurements show very little change compared to the ground state 2 ps after photoexcitation.^34^ The ground state is *S* = 6,^35,46^ and so consequently excitation at 400 nm (3.1 eV) will bring the system into a dense manifold of excited states (Table S2) coupled by the spin–orbit interaction, which must have a spin quantum number equal to or less than *S* = 6, reducing the effective spin state and red shifting the spectrum before structural changes dominate. At longer times, the system has returned to the ground state but is vibrationally hot. This excess energy will modify the relative population of the low-lying spin states (Table S2) until vibrational cooling occurs.

To gain more insight into the kinetics of the photoinduced dynamics, time delay scans were carried out with a step size of 100 fs up to 1500 fs for Mn_3_. Kinetic traces of selected Kα and Kβ probe energies are shown in Figures 4b and 4d, respectively. Interestingly, the Kα_1_ kinetic traces (5.8850 and 5.8885 keV) are more sensitive to a sub-100 fs decay than the Kα_2_ kinetics (5.8980 and 5.9010 keV). The high-energy edge of the Kβ_1,3_ peak initially shows a fast decrease in intensity but then increases again, which can also be observed in the difference spectra shown in Figure 2d. The Kβ’ kinetics shows a delayed growth by around 150 fs with respect to the Kβ_1,3_ dynamics.

**Figure 4:**
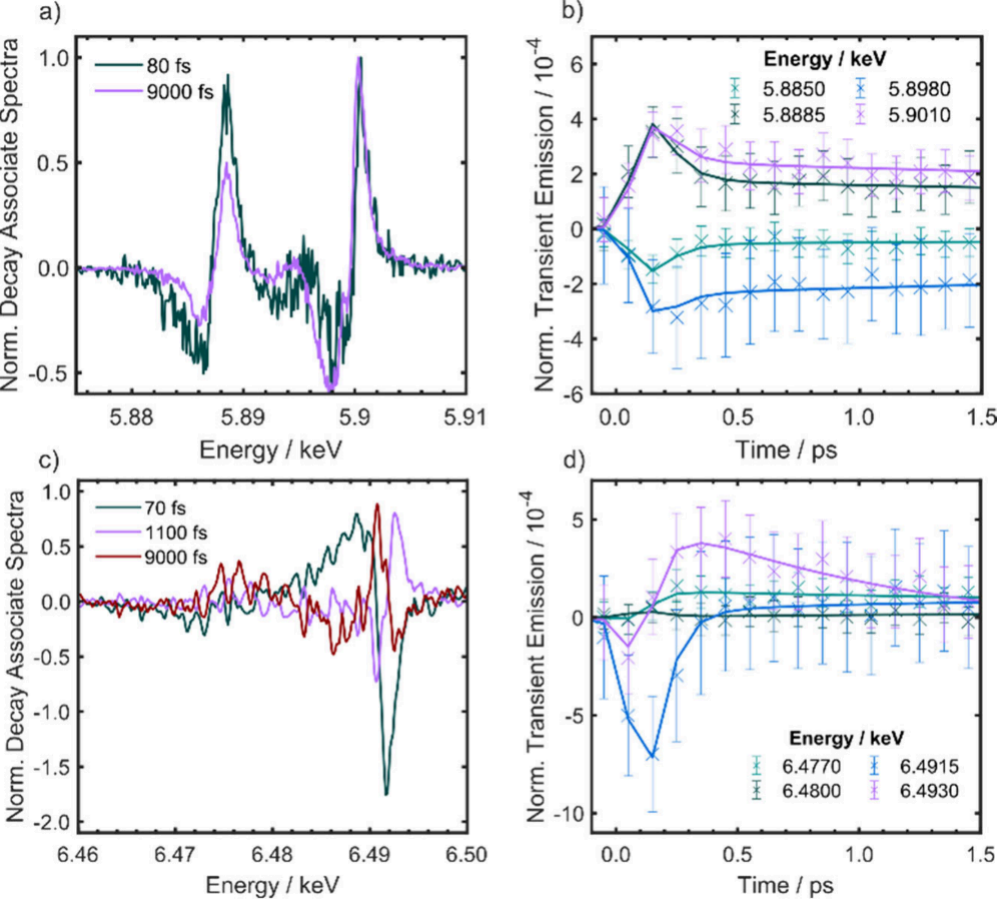
Global analysis of the TR-XES of Mn_3_. a) Decay-associated spectra of Kα. b) Time-domain fits of the Kα kinetic data from the global analysis. These have been normalized with respect to the ground state spectrum. c) Decay-associated spectra of Kβ. The Kβ decay-associated spectra were smoothed with a five-point Gaussian window. d) Time-domain fits of the Kβ kinetic data from the global analysis. The error bars describe the standard error of the mean (66% confidence interval).

To gain additional insight into the K-edge emission changes, global analysis was performed using the Python package, KiMoPack^47^ for the Mn_3_ data set. Global analysis has been used previously with X-ray emission data when there is a lack of suitable reference spectra for different spin states.^39^ Given the reduced signal-to-noise in the Mn(acac)_3_ data, we were not able to confidently model this data. A parallel kinetic model where each decay component occurs simultaneously was used to fit the data in Mn_3_, and the results are shown in Figure 4. Further details and additional models that were tested are discussed in the Supporting Information. The Kα emission could be modeled using two components with time constants of 80 fs (95% CI 40–250 fs) and 9000 fs, which is fixed to the longest-lived time component in the optical measurements.^30^ 80 fs is a smaller time constant than what was observed in the optical^30^ and X-ray absorption^34^ of 180 fs, which was attributed to internal conversion to the lowest energy excited state; however, it does lie within the 95% confidence intervals. Additionally, given that the instrument response function determined by reference measurements is 120 fs (see Supporting Information, Figure S6), the exact value of 80 fs is not physically meaningful. However, given the difference between the spectra at 50 and 200 fs there is clearly a distinct process occurring at these short time scales. The decay-associated spectra are plotted in Figure 4a. Both spectra are associated with a blue shift of the emission with respect to the ground state, consistent with the change in the effective nuclear charge discussed above.

In the 9000 fs decay-associated spectral component, the Kα_2_ signal is half as intense as the Kα_1_ signal. This is expected because of the degeneracy of the 2*p*_3/2_ and 2*p*_1/2_ orbitals (Figure 1a). A blue shift of the same energy across the spectrum would lead to the difference signal, where the Kα_1_ signal is twice as intense as the Kα_2_ signal, which is indeed observed in the 9000 fs decay-associated spectrum. Interestingly, both the Kα_1_ and Kα_2_ transitions in the 80 fs decay-associated spectrum have the same intensities. Given that the populations of the 2*p* orbitals do not change over the course of the dynamics, the stronger Kα_2_ difference signal in the 80 fs decay-associated spectrum suggests a larger blue shift than that of the Kα_1_ transition. Therefore, the splitting between the Kα transitions decreases suggesting a lower spin–orbit coupling. This agrees well with the early time Kβ difference spectrum, which suggests a reduction in *S*. However, additional effects may contribute to this observation such as changes in the superexchange interaction and mixing with valence orbitals.

The same analyses were carried out for the Kβ emission of Mn_3_. To achieve a satisfactory fit to the data, an additional exponential component was required. The fit yielded time constants of 70 fs (95% CI 30–650 fs), 1100 fs (95% CI 300–4900 fs) and 9000 fs, which was fixed. The decay-associated spectra are shown in Figure 4c, and fitted traces to selected probe energies are shown in Figure 4d. The 70 fs time constant agrees well with that found in the Kα fit. The 1100 fs component is consistent with a 1800 fs component that was identified in the optical transient absorption data as vibrational relaxation in the lowest energy excited state.^30^

The presented Mn_3_ TR-XES results lead us to reassess the assignments of time constants made using the optical measurements.^30^ The decay-associated spectrum of the 1100 fs time constant shares the blue shift of the XES in Figure 3d, which is calculated based on the structural change from the ground state to the minimum of the excited state established from X-ray absorption measurements.^34^ The decay of this spectral component is consistent with a change from the excited state geometry to a geometry more closely aligned with that of the ground state. Therefore, we reassign the 1–2 ps time constant observed in the Kβ and optical spectroscopy^30^ to internal conversion back to the ground state. Indeed, the transient signal after 2 ps is very weak and characterized by a small red shift indicative of a hot, highly mixed ground state with a lower average spin than the cooled ground state. This is corroborated by the time-resolved K-edge pre-edge absorption spectra, which are very similar to the ground state 2 ps after photoexcitation.^34^ Therefore, the 9000 fs component observed in the optical transient absorption and fixed in the fitting of the TR-XES data is assigned to cooling of the hot ground state. Although XES is not inherently sensitive to vibrational cooling, in these exchange-coupled complexes the energy spacing of the spin states is lower than that of typical vibrational modes (<100 cm^–1^). These have been calculated and are presented in Table S2. Therefore, cooling will also occur through different spin states, which XES is sensitive to.

Time-resolved X-ray K-edge emission spectroscopy has been used to study the dynamics in a trinuclear exchange-coupled SMM and a model monomeric complex to gain insight into the ultrafast spin dynamics that ensue after photoexcitation. In the monomeric Mn(acac)_3_, the Jahn–Teller switch from axial to equatorial elongation leads to an increase in the electron density on the Mn ion. This is reflected in a blue shift in the Kα spectrum. The Kβ spectrum exhibits a weaker signal, but the data between 300–700 fs can also be modeled by considering the same change in Jahn–Teller distortion which describes the Kα spectrum. The Kα spectrum of Mn_3_ exhibits the same blue shift as that of Mn(acac)_3_, which suggests an increase in metal charge density. This observation is supported by complementary NEVPT2 calculations. In contrast, the time-resolved Kβ emission of Mn_3_ is significantly more complex, which, owing to its increased sensitivity to the exchange interaction, suggests the involvement of multiple spin states in addition to the ground state, which has a spin quantum number of *S* = 6. At early times (∼50 fs after photoexcitation), before significant structural distortion, the system exists at a density of excited spin states. The total spin cannot exceed the *S* = 6 of the ground state^35^ and consequently any reduction in *S* leads to a red shift of the Kβ_1,3_ peak. After structural relaxation in the lowest excited state,^34^ the spectrum exhibits a blue shift, which is also observed in simulations of the X-ray spectrum based on structural changes. Finally, at longer times (>2 ps), the molecule has returned to a vibrationally hot ground state. While the ground state is formally *S* = 6, the high effective temperature will alter the equilibrium between the high density of spin states. This leads to another highly mixed state with a spin quantum number of less than *S* = 6 and another red shift (albeit smaller than the shift at early times) of the Kβ_1,3_ peak. A schematic of the dynamics occurring in Mn_3_ is shown in Figure 5.

**Figure 5:**
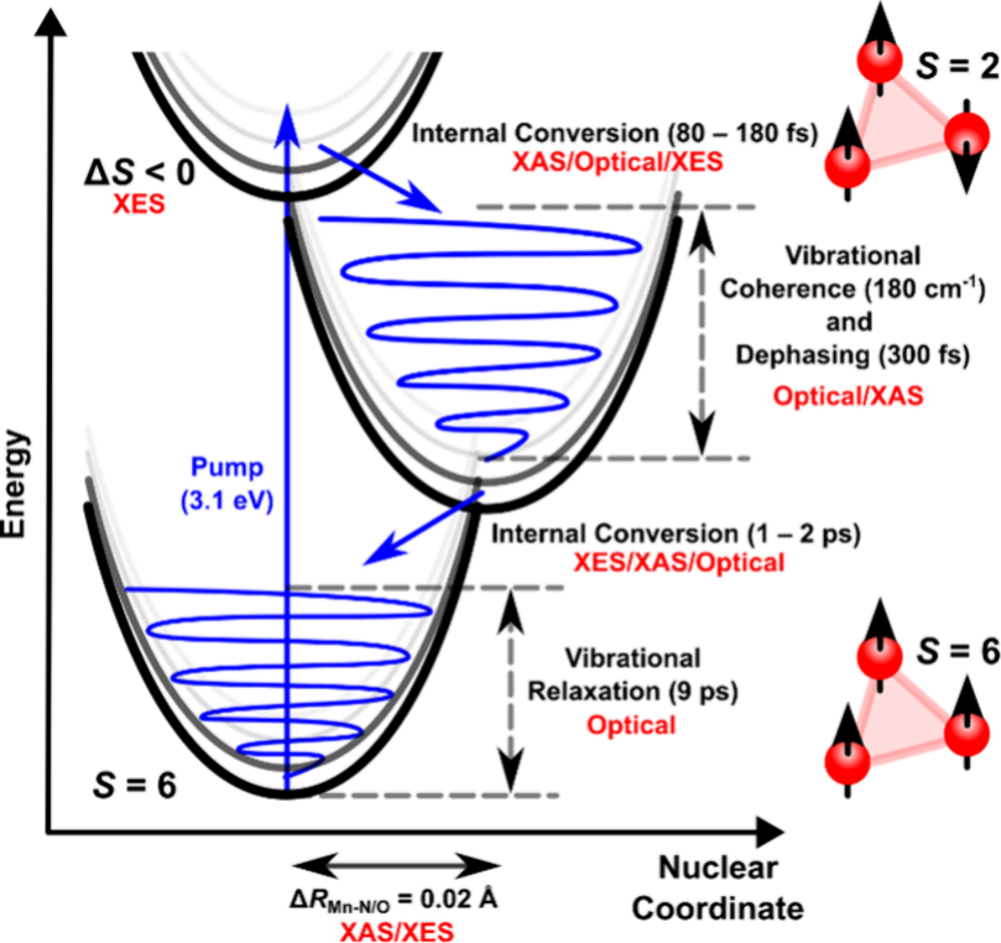
Scheme of the dynamics in Mn_3_. The text in red indicates if the time scales and assignments come from the time-resolved optical,^30^ X-ray absorption (XAS),^34^ and/or X-ray emission spectroscopy. The spin ground state and one of many possible excited-state spin configurations of Mn_3_ are shown at the right of the figure.

The results presented here show that the dynamics in polynuclear exchange-coupled transition metal complexes involve a complex interplay between spin, electronic, and nuclear degrees of freedom. However, using a combination of different ultrafast techniques, it is possible to decouple the effects of these processes and gain a deeper understanding. Indeed, by comparing to previous optical^30^ and X-ray absorption^34^ data, we have managed to separate the nuclear motion and changes in spin state in the TR-XES measurements in Mn_3_. Additionally, the combination of multiple ultrafast techniques in the optical and X-ray regimes has removed some of the ambiguity in the assignments of dynamical processes. Therefore, using a wide range of probes across the electromagnetic spectrum is important to the study of complex molecules, such as SMMs and more widely studied polynuclear transition metal complexes.

## Supporting Information Available

Experimental and computational methods, additional computational results and additional global analysis models. (PDF)
Transparent Peer Review report available (PDF)
