# Supplementary material for: Capturing Ultrafast Spin Dynamics in Single-Molecule Magnets Using Femtosecond X-ray Emission Spectroscopy
Source: J Phys Chem Lett. 2025 Apr 17;16(17):4148–54. doi: 10.1021/acs.jpclett.5c00383 (PMC12051188; doi:10.1021/acs.jpclett.5c00383)
Supplement: Supplementary file 1 — jz5c00383_si_001.pdf [file jz5c00383_si_001.pdf]

# Supporting information for 'Capturing Ultrafast Spin Dynamics in Single-molecule Magnets using Femtosecond X-ray Emission Spectroscopy'

Kyle Barlow,[1]* Ryan Phelps,[1] Julien Eng,[2] Rebecca A. Ingle,[3] Dmitry Khakhulin,[4] Mykola Biednov,[4] Sharmistha Paul Dutta,[4] Yifeng Jiang,[4] Frederico A. Lima,[4] Vandana Tiwari,[4] Christopher Milne,[4] Tetsuo Katayama,[5,6] Marco Coletta,[1] Euan K. Brechin,[1] Thomas J. Penfold,[2]* J. Olof Johansson[1]*

*[1]EaStCHEM School of Chemistry, University of Edinburgh, David Brewster Road, EH9 3FJ, Edinburgh, UK.*

*[2]Chemistry, School of Natural and Environmental Sciences, Newcastle University, Newcastle upon Tyne, UK*

*[3] Department of Chemistry, University College London, 20 Gordon Street, London, WC1H 0AJ, UK*

*[4]European XFEL GmbH Holzkoppel 4, 22869 Schenefeld, Germany*

*[5]Japan Synchrotron Radiation Research Institute, Kouto 1-1-1, Sayo, Hyogo 679-5198, Japan.*

*[6]RIKEN SPring-8 Center, 1-1-1 Kouto, Sayo, Hyogo 679-5148, Japan.*

## Supplementary Methods

### *Sample Preparation and Delivery*

Ethanoic solutions of $Mn(acac)_3$ (purchased from Sigma-Aldrich and used without further purification) and $Mn_3$ (synthesised as previously described[1]) were prepared with concentrations of 60 mM and 5 mM, respectively. The sample was circulated through a HPLC pump-based system through a glass capillary nozzle that created a circular jet of 100 µm diameter within a He environment with 25 µm thick Kapton windows. The speed of the jet was around 60 $ms^{-1}$, which replenishes the sample between every X-ray pulse. No degradation of the sample was observed via *in situ* UV-Vis spectrometer or changes in the K-edge emission over time. The sample concentration was kept constant by slowly refilling the sample in the case of solvent evaporation.

### *X-ray Spectroscopy*

The X-ray experiments were carried out at the Femtosecond X-ray Experiments (FXE) beamline at the European X-ray free electron laser (EU-XFEL). The K-edge X-ray emission was collected with a Von Hamos spectrometer which was equipped with cylindrical crystal analysers with a 50 cm radius. Five Ge(111) crystals were used to collect the Mn Kα emission and eight Ge(110) analysers for the Mn Kβ emission. The spectra were focussed onto the same Jungfrau-500k detector allowing simultaneous and correlated detection of both emission lines. Figure S1 shows a typical Jungfrau image with Kα and Kβ emission highlighted. To calibrate the horizontal pixels to absolute energy the $K\alpha_1$, $K\alpha_2$, $K\beta_{1,3}$ and Kβ' maxima were compared to literature values[2,3] and the conversion set accordingly.

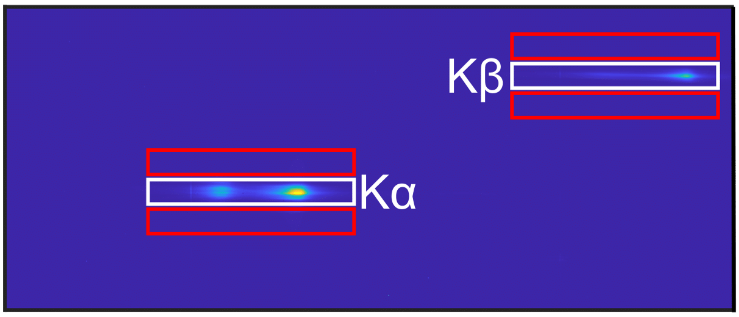


**Figure S1.** Typical Jungfrau image of the Mn Kα and Kβ emission. The white boxes are integrated along the vertical axis to extract the spectra. The red boxes are integrated in the vertical direction and subtracted to correct for any background.

The TRXES experiments used 7.5 keV X-ray photons which are bunched into individual trains of 86 pulses with an intratrain repetition rate of 282 kHz and intertrain rate of 10 Hz. The X-ray pulse was focussed to a beam diameter of around 7 µm (FWHM). The optical pump laser wavelength was set to 400 nm, focussed to a 60 µm (FWHM) spot size and overlapped with the sample and X-ray pulses. 400 nm corresponds to the same optical transition in $Mn(acac)_3$ and $Mn_3$.[4] The optical spectra are shown in Figure S2. The repetition rate of the optical laser matched the intratrain repetition rate the X-ray at 282 kHz. The optical laser was chopped at 5 Hz to measure a pump-on and pump-off train consecutively. The signal is integrated across an entire train (around 350 µs), therefore an X-ray emission spectrum is measured every 100 ms for all 86 X-ray pulses in a train.

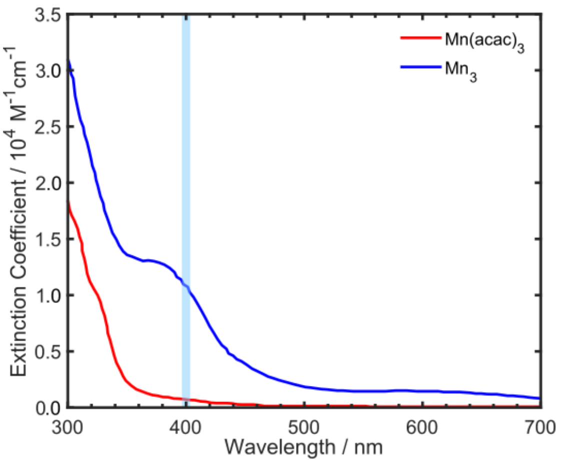


**Figure S2.** Optical absorption spectrum of ethanoic solutions of $Mn(acac)_3$ and $Mn_3$. The pump is indicated by a light blue line at 400 nm. Data from ref. [4].

The X-ray emission spectra imaged by the Jungfrau detector were corrected for the gain of each pixel after removing the respective electronic offset according to a standard procedure.[5] To extract the 1D Kα and Kβ spectra, around 10–15 vertical pixels around the region of interest (white boxes, Fig S1) were averaged. A background was calculated by averaging the regions vertical pixels either side of the spectrum (red boxes, Fig S1). This background was subtracted from the spectra assuming it yields a good approximation of the elastic scattering background.

To calculate a transient spectrum, each spectrum was self-normalised to the area under the curve (to correct for fluctuations in X-ray intensities, pointing instabilities, etc.) and every laser on and laser off spectrum was averaged. The laser off spectrum were subtracted from the laser on spectrum yielding a difference spectrum. A difference spectrum is always a function of some experimental parameter(s), here they are the pump-probe time delay and excitation fluence.

For scans as a function of excitation fluence, the Kα spectra are plotted and scaled with their respective energies for $Mn(acac)_3$ and $Mn_3$ (Figures S3a and S3c). The integrated intensity of the $K\alpha_1$ transient is also plotted as a function of pulse energy (Figures S3b and S3d). The excitation energy was chosen in the linear excitation regime. This corresponds to a pulse energy of around 6.9 µJ (130 mJ/cm$^2$) and 14 µJ (260 mJ/cm$^2$) for $Mn_3$ and $Mn(acac)_3$, respectively. Given these pump fluences and taking into account the concentration, path length and extinction coefficient at the pump wavelengths of the samples, it suggests $Mn(acac)_3$ absorbs 1 photon per molecule and $Mn_3$ absorbs 6.6 photons per molecule.

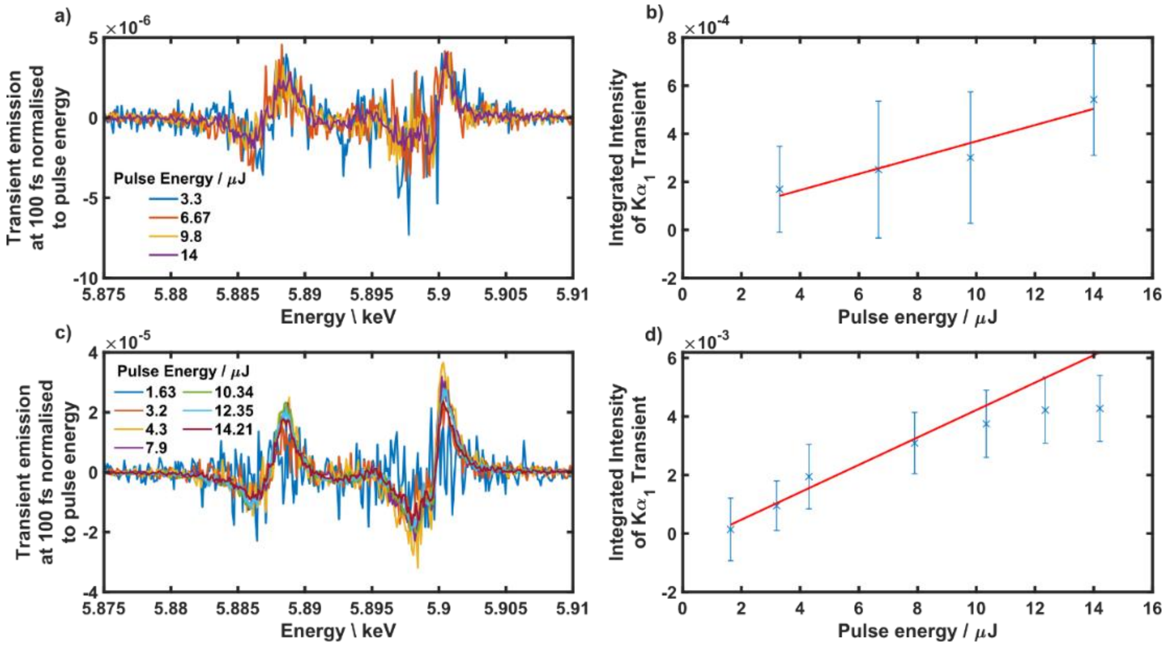


**Figure S3.** Power dependence of transient signal. a) Transient Kα spectrum of $Mn(acac)_3$ at different excitation powers. The spectra have been normalised to the pulse energy. b) Integrated intensity of the high-energy part of the $K\alpha_1$ transient of $Mn(acac)_3$ as a function of pump pulse energy fitted with a straight line. c) Transient Kα spectrum of $Mn(acac)_3$ at different excitation powers. The spectra have been normalised to the pulse energy. d) Integrated intensity of the high-energy part of the $K\alpha_1$ transient of $Mn_3$ as a function of pump pulse energy fitted with a straight line up to 8 µJ. The plot loses linearity around this value.

For measurements as a function of pump-probe delay time, the signal was typically integrated for around 30–60 minutes for a single fixed time point measurement. For kinetic scans (pump-probe delay time is changed in a stepwise fashion) each 50 fs time point was integrated for around 2 minutes and binned into 100 fs bins.

*Excited State Computational Methods*

Electronic structure calculations were performed using the ORCA 4 quantum chemistry package.[6,7]

$Mn(acac)_3$ and $[Mn_3O(Et\text{-}sao)_3(\beta\text{-}pic)_3(ClO_4)]$ ground state geometries were optimised using the density functional theory (DFT) approach with the PBE0 functional[8] and the def2-TZVP basis set[9] and def2-SV(P),[10] respectively.

Electronic structure was computed at each optimised geometry through the state-average complete active space self-consistent method (SA-CASSCF) method with dynamic correlation added through the N-electron valence perturbation theory at the second order (NEVPT2) method using an active space comprising 4 electrons in the five *d*-orbitals of the manganese atom for $Mn(acac)_3$ (Figure S4) and 12 electrons in the 15 *d*-orbitals of the three manganese atoms for $[Mn_3O(Et\text{-}sao)_3(\beta\text{-}pic)_3(ClO_4)]$ (Figure S5). The same basis sets as for the optimisation have been employed for consistency. Relativistic effects were included using the Douglas Kroll Hess approach at the second order operator.[10–13]

All minimum energy geometries were confirmed by frequency analysis at the same level of theory.

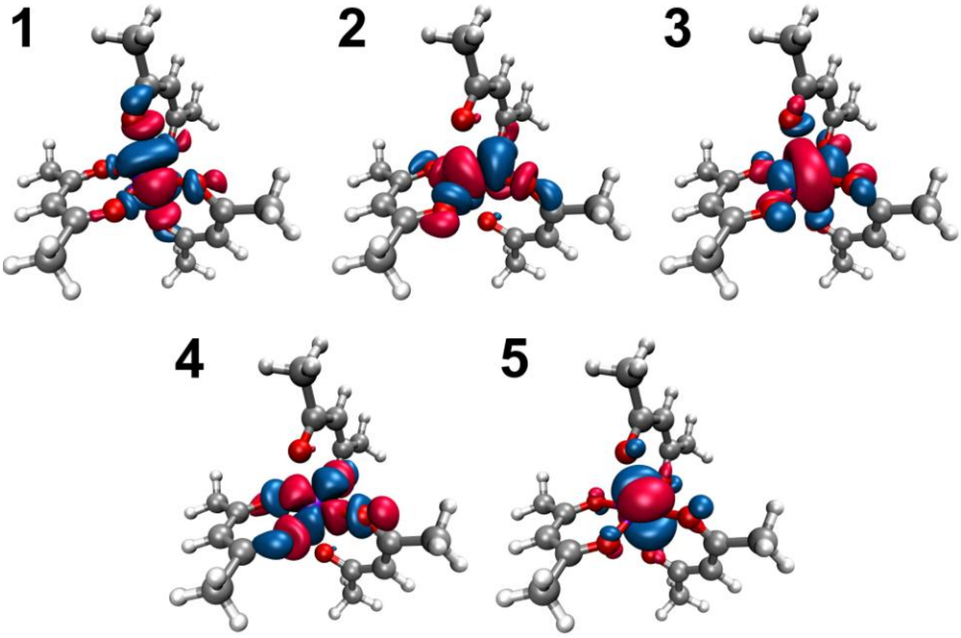

**Figure S4.** Orbitals included in the active space of Mn(acac)$_3$.

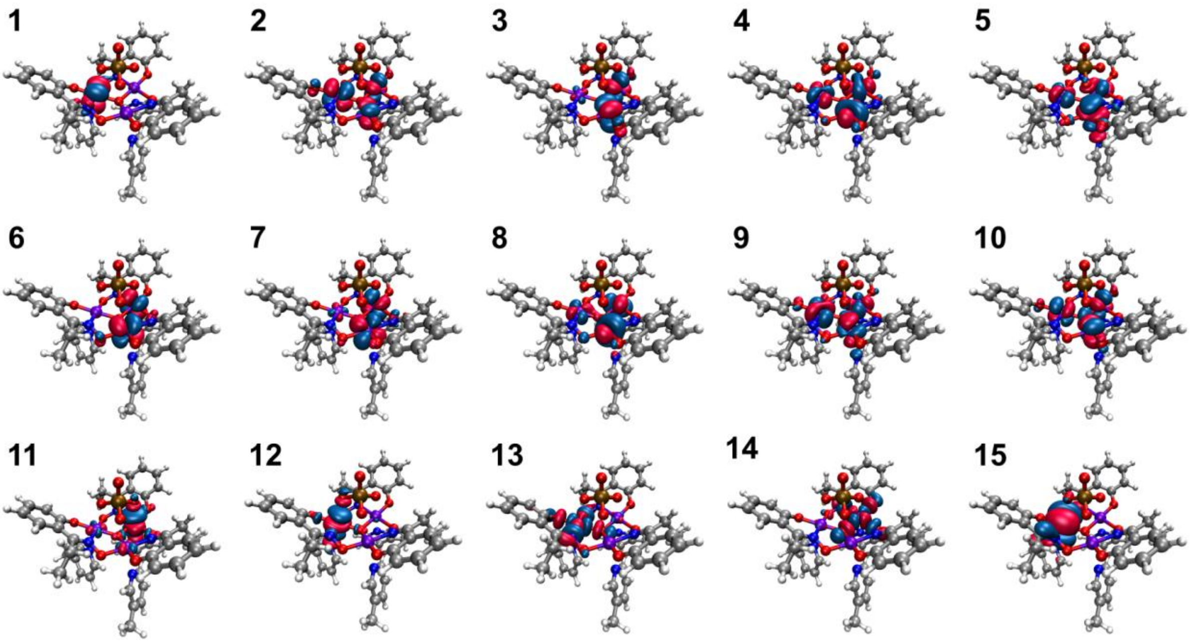

**Figure S5**. Orbitals included in the active space of [Mn$_3$O(Et-sao)$_3$(β-pic)$_3$(ClO$_4$)].

*X-ray Emission Computational Methods*

The X-ray emission simulations were performed using the ORCA 4 quantum chemistry package.[6,7] Using the structures from the geometry optimisations, a state-average complete active space self-consistent method (SA-CASSCF) calculations was performed using an active space comprising 4 electrons in the 5 *d*-orbitals of the manganese atom. This calculation included 5 quintet state, 45 triplet states and 50 singlet states. Following the initial SA-CASSCF simulation, we performed a RASSF simulations using this SA-CASSCF simulation as a reference. The total active space was extended by 6 orbitals (1s core and 2p or 3p core orbitals) and 7 electrons. These orbitals were incorporated into the RAS1 space, ensuring a maximum of one hole, which one enforced by increasing the charge of the complex to reflect the initial core excitation.

## Supplementary Results

*Computational Excited State Simulations*

Tables S1 and S2 show the calculated states using SOC-NEVPT2 for Mn(acac)$_3$ and [Mn$_3$O(Et-sao)$_3$(β-pic)$_3$(ClO$_4$)], respectively.

| | | | **Composition** | |
|---|---|---|---|---|
| **State** | **ΔE / eV** | **% S=2** | **% S=1** | **% S=0** |
| 1 | 0.000 | 100 | 0 | 0 |
| 2 | 0.000 | 100 | 0 | 0 |
| 3 | 0.002 | 100 | 0 | 0 |
| 4 | 0.002 | 100 | 0 | 0 |
| 5 | 0.002 | 100 | 0 | 0 |
| 6 | 1.186 | 97 | 0 | 0 |
| 7 | 1.187 | 97 | 0 | 0 |
| 8 | 1.187 | 98 | 1 | 0 |
| 9 | 1.191 | 98 | 0 | 0 |
| 10 | 1.191 | 98 | 1 | 0 |
| 11 | 1.347 | 2 | 97 | 0 |
| 12 | 1.349 | 0 | 99 | 0 |
| 13 | 1.355 | 0 | 98 | 0 |
| 14 | 1.396 | 0 | 98 | 0 |
| 15 | 1.403 | 2 | 98 | 0 |
| 16 | 1.404 | 0 | 98 | 0 |
| 17 | 1.583 | 0 | 99 | 0 |
| 18 | 1.583 | 0 | 99 | 0 |
| 19 | 1.585 | 0 | 98 | 0 |
| 20 | 2.525 | 99 | 0 | 0 |
| 21 | 2.526 | 99 | 0 | 0 |
| 22 | 2.526 | 99 | 0 | 0 |
| 23 | 2.528 | 99 | 0 | 0 |
| 24 | 2.528 | 99 | 0 | 0 |
| 25 | 2.665 | 2 | 68 | 29 |
| 26 | 2.669 | 0 | 78 | 21 |
| 27 | 2.679 | 0 | 97 | 1 |
| 28 | 2.712 | 0 | 34 | 64 |
| 29 | 2.717 | 5 | 24 | 69 |
| 30 | 2.737 | 84 | 12 | 1 |
| 31 | 2.739 | 87 | 7 | 3 |
| 32 | 2.742 | 85 | 7 | 6 |

| | | | | |
|---|---|---|---|---|
| 33 | 2.745 | 95 | 3 | 0 |
| 34 | 2.745 | 95 | 0 | 0 |
| 35 | 2.810 | 28 | 43 | 27 |
| 36 | 2.819 | 32 | 48 | 18 |
| 37 | 2.828 | 22 | 30 | 46 |
| 38 | 2.830 | 40 | 56 | 1 |
| 39 | 2.849 | 89 | 9 | 0 |
| 40 | 2.852 | 89 | 9 | 0 |
| 41 | 2.870 | 67 | 24 | 8 |
| 42 | 2.870 | 70 | 28 | 0 |
| 43 | 2.874 | 64 | 32 | 2 |
| 44 | 2.883 | 0 | 23 | 74 |
| 45 | 2.944 | 3 | 96 | 0 |
| 46 | 2.952 | 2 | 96 | 0 |
| 47 | 2.958 | 0 | 86 | 10 |
| 48 | 2.986 | 7 | 90 | 2 |
| 49 | 2.994 | 8 | 90 | 0 |

**Table S1.** Every spin state and their spin character < 3.0 eV for $Mn(acac)_3$ calculated using NEVPT2 including SOC at the ground state geometry. The contribution of different spin components is given in %.

| | | Contributions | | | | | | |
|---|---|---|---|---|---|---|---|---|
| State | ΔE / eV | % S=6 | % S=5 | % S=4 | % S=3 | % S=2 | % S=1 | % S=0 |
| 1 | 0.000 | 0 | 0 | 0 | 0 | 0 | 5 | 93 |
| 2 | 0.000 | 0 | 0 | 0 | 0 | 0 | 97 | 3 |
| 3 | 0.000 | 0 | 0 | 0 | 0 | 0 | 98 | 0 |
| 4 | 0.001 | 0 | 0 | 0 | 0 | 0 | 98 | 0 |
| 5 | 0.001 | 0 | 0 | 0 | 0 | 0 | 97 | 0 |
| 6 | 0.001 | 0 | 0 | 0 | 0 | 0 | 98 | 2 |
| 7 | 0.001 | 0 | 0 | 0 | 0 | 0 | 98 | 0 |
| 8 | 0.001 | 0 | 0 | 0 | 0 | 6 | 89 | 2 |
| 9 | 0.001 | 0 | 0 | 0 | 0 | 3 | 94 | 0 |
| 10 | 0.001 | 0 | 0 | 0 | 0 | 2 | 95 | 0 |
| 11 | 0.001 | 0 | 0 | 0 | 0 | 98 | 0 | 0 |
| 12 | 0.001 | 0 | 0 | 0 | 0 | 98 | 0 | 0 |
| 13 | 0.001 | 0 | 0 | 0 | 0 | 96 | 0 | 0 |
| 14 | 0.001 | 0 | 0 | 0 | 0 | 97 | 0 | 0 |
| 15 | 0.001 | 0 | 0 | 0 | 0 | 98 | 1 | 0 |
| 16 | 0.001 | 0 | 0 | 0 | 0 | 99 | 0 | 0 |
| 17 | 0.001 | 0 | 0 | 0 | 0 | 99 | 0 | 0 |
| 18 | 0.001 | 0 | 0 | 0 | 0 | 93 | 2 | 0 |
| 19 | 0.001 | 0 | 0 | 0 | 0 | 93 | 3 | 0 |
| 20 | 0.001 | 0 | 0 | 0 | 4 | 92 | 0 | 0 |
| 21 | 0.001 | 0 | 0 | 0 | 0 | 92 | 5 | 0 |
| 22 | 0.001 | 0 | 0 | 0 | 3 | 92 | 0 | 0 |
| 23 | 0.001 | 0 | 0 | 0 | 3 | 92 | 0 | 0 |
| 24 | 0.001 | 0 | 0 | 0 | 8 | 89 | 0 | 0 |
| 25 | 0.001 | 0 | 0 | 0 | 8 | 89 | 0 | 0 |
| 26 | 0.001 | 0 | 0 | 0 | 98 | 0 | 0 | 0 |
| 27 | 0.001 | 0 | 0 | 0 | 98 | 0 | 0 | 0 |

| | | | | | | | | |
|---|---|---|---|---|---|---|---|---|
| 28 | 0.001 | 84 | 0 | 8 | 0 | 0 | 0 | 0 |
| 29 | 0.001 | 84 | 0 | 8 | 0 | 0 | 0 | 0 |
| 30 | 0.001 | 0 | 0 | 0 | 90 | 5 | 0 | 0 |
| 31 | 0.001 | 0 | 0 | 0 | 90 | 6 | 0 | 0 |
| 32 | 0.001 | 0 | 0 | 0 | 93 | 3 | 0 | 0 |
| 33 | 0.001 | 0 | 0 | 0 | 93 | 4 | 0 | 0 |
| 34 | 0.001 | 0 | 0 | 0 | 94 | 3 | 0 | 0 |
| 35 | 0.001 | 82 | 4 | 9 | 0 | 0 | 0 | 0 |
| 36 | 0.001 | 80 | 4 | 7 | 0 | 0 | 0 | 0 |
| 37 | 0.001 | 78 | 5 | 12 | 0 | 0 | 0 | 0 |
| 38 | 0.001 | 71 | 11 | 7 | 0 | 0 | 0 | 0 |
| 39 | 0.001 | 70 | 10 | 10 | 0 | 0 | 0 | 0 |
| 40 | 0.001 | 0 | 0 | 5 | 93 | 0 | 0 | 0 |
| 41 | 0.001 | 0 | 0 | 5 | 93 | 0 | 0 | 0 |
| 42 | 0.001 | 36 | 21 | 7 | 28 | 0 | 0 | 0 |
| 43 | 0.001 | 38 | 21 | 6 | 25 | 0 | 0 | 0 |
| 44 | 0.001 | 3 | 0 | 12 | 73 | 0 | 0 | 0 |
| 45 | 0.001 | 12 | 4 | 11 | 58 | 0 | 0 | 0 |
| 46 | 0.001 | 0 | 0 | 26 | 66 | 0 | 0 | 0 |
| 47 | 0.001 | 9 | 3 | 23 | 56 | 0 | 0 | 0 |
| 48 | 0.001 | 4 | 3 | 36 | 47 | 0 | 0 | 0 |
| 49 | 0.001 | 0 | 0 | 99 | 0 | 0 | 0 | 0 |
| 50 | 0.001 | 0 | 0 | 99 | 0 | 0 | 0 | 0 |
| 51 | 0.001 | 0 | 0 | 91 | 6 | 0 | 0 | 0 |
| 52 | 0.001 | 0 | 0 | 91 | 6 | 0 | 0 | 0 |
| 53 | 0.001 | 0 | 0 | 71 | 24 | 0 | 0 | 0 |
| 54 | 0.001 | 0 | 0 | 73 | 22 | 0 | 0 | 0 |
| 55 | 0.001 | 0 | 0 | 13 | 75 | 2 | 0 | 0 |
| 56 | 0.001 | 0 | 0 | 43 | 48 | 0 | 0 | 0 |
| 57 | 0.001 | 0 | 0 | 33 | 58 | 0 | 0 | 0 |
| 58 | 0.001 | 33 | 57 | 0 | 0 | 0 | 0 | 0 |
| 59 | 0.001 | 31 | 58 | 0 | 1 | 0 | 0 | 0 |
| 60 | 0.001 | 0 | 0 | 57 | 39 | 0 | 0 | 0 |
| 61 | 0.001 | 0 | 0 | 27 | 64 | 0 | 0 | 0 |
| 62 | 0.001 | 0 | 0 | 26 | 64 | 0 | 0 | 0 |
| 63 | 0.001 | 0 | 0 | 6 | 84 | 0 | 0 | 0 |
| 64 | 0.001 | 0 | 0 | 6 | 84 | 0 | 0 | 0 |
| 65 | 0.001 | 0 | 0 | 0 | 94 | 0 | 0 | 0 |
| 66 | 0.001 | 0 | 0 | 0 | 94 | 0 | 0 | 0 |
| 67 | 0.002 | 3 | 5 | 88 | 0 | 0 | 0 | 0 |
| 68 | 0.002 | 3 | 5 | 88 | 0 | 0 | 0 | 0 |
| 69 | 0.002 | 0 | 11 | 81 | 0 | 0 | 0 | 0 |
| 70 | 0.002 | 0 | 11 | 81 | 0 | 0 | 0 | 0 |
| 71 | 0.002 | 0 | 18 | 73 | 0 | 0 | 0 | 0 |
| 72 | 0.002 | 0 | 18 | 73 | 0 | 0 | 0 | 0 |
| 73 | 0.002 | 0 | 22 | 71 | 0 | 0 | 0 | 0 |
| 74 | 0.002 | 0 | 28 | 68 | 0 | 0 | 0 | 0 |
| 75 | 0.002 | 0 | 26 | 70 | 0 | 0 | 0 | 0 |
| 76 | 0.002 | 0 | 96 | 0 | 0 | 0 | 0 | 0 |
| 77 | 0.002 | 0 | 96 | 0 | 0 | 0 | 0 | 0 |
| 78 | 0.002 | 0 | 90 | 3 | 0 | 0 | 0 | 0 |
| 79 | 0.002 | 0 | 90 | 3 | 0 | 0 | 0 | 0 |
| 80 | 0.002 | 0 | 84 | 11 | 0 | 0 | 0 | 0 |
| 81 | 0.002 | 0 | 84 | 11 | 0 | 0 | 0 | 0 |

| | | | | | | | | |
|---|---|---|---|---|---|---|---|---|
| 82 | 0.002 | 0 | 77 | 16 | 0 | 0 | 0 | 0 |
| 83 | 0.002 | 0 | 76 | 18 | 0 | 0 | 0 | 0 |
| 84 | 0.002 | 0 | 70 | 25 | 0 | 0 | 0 | 0 |
| 85 | 0.002 | 0 | 68 | 27 | 0 | 0 | 0 | 0 |
| 86 | 0.002 | 0 | 66 | 30 | 0 | 0 | 0 | 0 |
| 87 | 0.002 | 2 | 0 | 85 | 0 | 0 | 0 | 0 |
| 88 | 0.002 | 0 | 0 | 84 | 0 | 0 | 0 | 0 |
| 89 | 0.002 | 8 | 0 | 82 | 0 | 0 | 0 | 0 |
| 90 | 0.002 | 6 | 0 | 86 | 0 | 0 | 0 | 0 |
| 91 | 0.002 | 9 | 0 | 84 | 0 | 0 | 0 | 0 |
| 92 | 0.002 | 7 | 0 | 86 | 0 | 0 | 0 | 0 |
| 93 | 0.002 | 7 | 0 | 86 | 0 | 0 | 0 | 0 |
| 94 | 0.002 | 5 | 0 | 90 | 0 | 0 | 0 | 0 |
| 95 | 0.002 | 5 | 0 | 90 | 0 | 0 | 0 | 0 |
| 96 | 0.002 | 61 | 33 | 0 | 0 | 0 | 0 | 0 |
| 97 | 0.002 | 61 | 33 | 0 | 0 | 0 | 0 | 0 |
| 98 | 0.002 | 47 | 48 | 0 | 0 | 0 | 0 | 0 |
| 99 | 0.002 | 48 | 47 | 0 | 0 | 0 | 0 | 0 |
| 100 | 0.002 | 32 | 62 | 0 | 0 | 0 | 0 | 0 |
| 101 | 0.002 | 33 | 60 | 0 | 0 | 0 | 0 | 0 |
| 102 | 0.002 | 21 | 73 | 0 | 0 | 0 | 0 | 0 |
| 103 | 0.002 | 25 | 70 | 0 | 0 | 0 | 0 | 0 |
| 104 | 0.002 | 15 | 79 | 0 | 0 | 0 | 0 | 0 |
| 105 | 0.002 | 27 | 69 | 0 | 0 | 0 | 0 | 0 |
| 106 | 0.002 | 23 | 72 | 0 | 0 | 0 | 0 | 0 |
| 107 | 0.002 | 2 | 90 | 0 | 0 | 0 | 0 | 0 |
| 108 | 0.002 | 2 | 88 | 0 | 0 | 0 | 0 | 0 |
| 109 | 2.054 | 0 | 0 | 0 | 0 | 0 | 0 | 100 |
| 110 | 2.058 | 0 | 100 | 0 | 0 | 0 | 0 | 0 |
| 111 | 2.058 | 0 | 100 | 0 | 0 | 0 | 0 | 0 |
| 112 | 2.058 | 0 | 100 | 0 | 0 | 0 | 0 | 0 |
| 113 | 2.058 | 0 | 99 | 0 | 0 | 0 | 0 | 0 |
| 114 | 2.058 | 0 | 100 | 0 | 0 | 0 | 0 | 0 |
| 115 | 2.059 | 0 | 100 | 0 | 0 | 0 | 0 | 0 |
| 116 | 2.059 | 0 | 100 | 0 | 0 | 0 | 0 | 0 |
| 117 | 2.059 | 0 | 100 | 0 | 0 | 0 | 0 | 0 |
| 118 | 2.059 | 0 | 100 | 0 | 0 | 0 | 0 | 0 |
| 119 | 2.059 | 0 | 100 | 0 | 0 | 0 | 0 | 0 |
| 120 | 2.059 | 0 | 100 | 0 | 0 | 0 | 0 | 0 |
| 121 | 2.580 | 0 | 0 | 0 | 0 | 0 | 0 | 100 |
| 122 | 2.613 | 99 | 0 | 0 | 0 | 0 | 0 | 0 |
| 123 | 2.613 | 99 | 0 | 0 | 0 | 0 | 0 | 0 |
| 124 | 2.613 | 98 | 0 | 0 | 0 | 0 | 0 | 0 |
| 125 | 2.613 | 100 | 0 | 0 | 0 | 0 | 0 | 0 |
| 126 | 2.613 | 99 | 0 | 0 | 0 | 0 | 0 | 0 |
| 127 | 2.613 | 100 | 0 | 0 | 0 | 0 | 0 | 0 |
| 128 | 2.613 | 100 | 0 | 0 | 0 | 0 | 0 | 0 |
| 129 | 2.613 | 100 | 0 | 0 | 0 | 0 | 0 | 0 |
| 130 | 2.613 | 100 | 0 | 0 | 0 | 0 | 0 | 0 |
| 131 | 2.613 | 100 | 0 | 0 | 0 | 0 | 0 | 0 |
| 132 | 2.614 | 100 | 0 | 0 | 0 | 0 | 0 | 0 |
| 133 | 2.614 | 100 | 0 | 0 | 0 | 0 | 0 | 0 |
| 134 | 2.614 | 100 | 0 | 0 | 0 | 0 | 0 | 0 |
| 135 | 2.659 | 99 | 0 | 0 | 0 | 0 | 0 | 0 |

| | | | | | | | | |
|---|---|---|---|---|---|---|---|---|
| 136 | 2.659 | 100 | 0 | 0 | 0 | 0 | 0 | 0 |
| 137 | 2.659 | 100 | 0 | 0 | 0 | 0 | 0 | 0 |
| 138 | 2.659 | 100 | 0 | 0 | 0 | 0 | 0 | 0 |
| 139 | 2.659 | 100 | 0 | 0 | 0 | 0 | 0 | 0 |
| 140 | 2.659 | 100 | 0 | 0 | 0 | 0 | 0 | 0 |
| 141 | 2.659 | 100 | 0 | 0 | 0 | 0 | 0 | 0 |
| 142 | 2.659 | 98 | 0 | 0 | 0 | 0 | 0 | 0 |
| 143 | 2.659 | 98 | 0 | 0 | 0 | 0 | 0 | 0 |
| 144 | 2.659 | 100 | 0 | 0 | 0 | 0 | 0 | 0 |
| 145 | 2.659 | 100 | 0 | 0 | 0 | 0 | 0 | 0 |
| 146 | 2.659 | 98 | 0 | 0 | 0 | 0 | 0 | 0 |
| 147 | 2.659 | 100 | 0 | 0 | 0 | 0 | 0 | 0 |

**Table S2.** Every spin state and their spin character < 3.0 eV for [$Mn_3O(Et\text{-}sao)_3(\beta\text{-}pic)_3(ClO_4)$] calculated using NEVPT2 including SOC at the ground state geometry. The contribution of different spin components is given in %.

## Modelling of TR-XES signals

*Determination of Instrument Response Function Width*

Kinetics models that were used to fit the data are convoluted with a Gaussian instrument response function ($\mathrm{IRF}(\sigma, t) = \frac{1}{\sqrt{2\pi}\sigma} e^{-\frac{t^2}{2\sigma^2}}$) with temporal width $\sigma$ and time $t$. The width of the IRF was determined by measuring the photoinduced change in the Kα emission of $[Fe(bpy)_3]^{2+}$ in ethanol after 400 nm excitation and globally fitting Equation S1 to the data with fixed $\tau$ = 126 fs and allowing time-zero ($t_0$) and the amplitudes to vary.[14] These experiments were performed immediately before those using the Mn-based samples and had the same jet thickness, optical laser and XFEL pulses. The fits are shown in Figure S6 and yielded $\sigma = 60 \pm 20$ fs, which is consistent with the FWHM (around 120 fs) of the IRF previously reported from studies at FXE.[14] Time-zero was found to be $t_0 = 180 \pm 60$ fs.

$$I(t) = \mathrm{IRF}(\sigma, (t - t_0)) * A(1 - e^{-\frac{(t-t_0)}{\tau}}) \qquad \text{Equation S1}$$

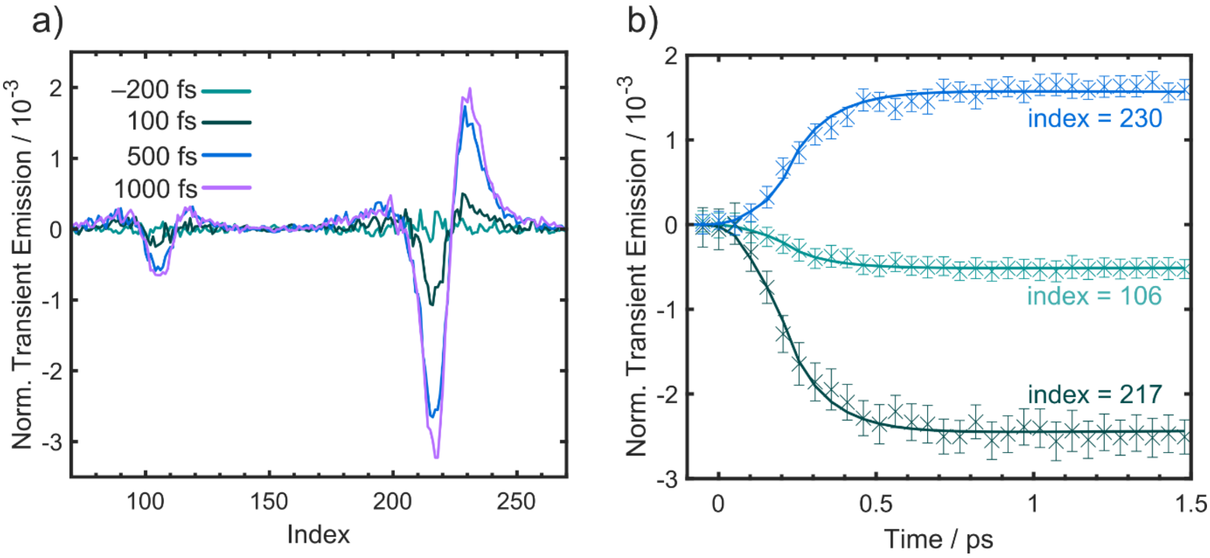


**Figure S6.** TR-XES of $[Fe(bpy)_3]^2$ to determine the IRF width. a) Transient difference emission spectra at different time delays after 400 nm photoexcitation of a 15 mM ethanoic solution of $[Fe(bpy)_3]^{2+}$. b) Global fitting results as a function of time for different X-ray probe energies using Equation S1.

*Modelling $Mn_3$ Kβ with only two exponential terms.*

Figure S7 displays the fitting results of a parallel global model using only two exponential components to the $Mn_3$ Kβ dataset. The fit yielded time constants of 60 fs (95 % CI 10 – 70 fs) and a second time constant of 9000 fs, which was fixed. This generally provides an adequate fit however, the ≈ 1 ps decay at 6.4930 keV is not captured. Additionally, the 50 fs time points in the 6.4915 and 6.4930 keV time trace are not modelled well.

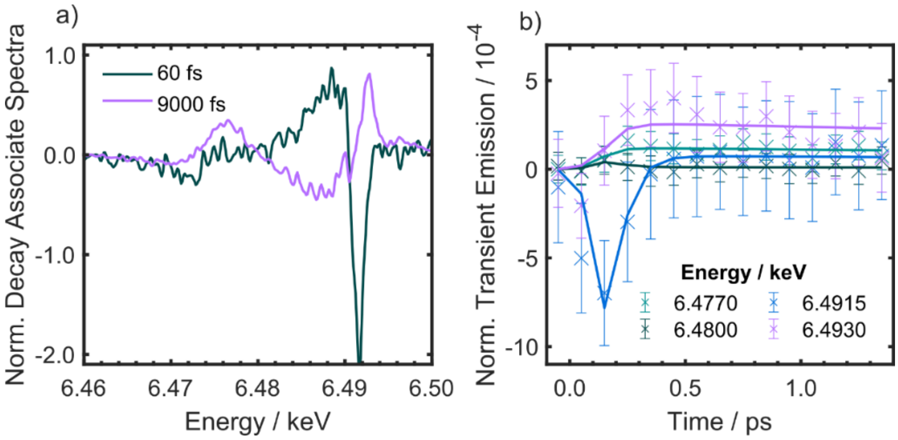


**Figure S7.** Global analysis of the Kβ TR-XES of $Mn_3$ using only two exponential components. a) Decay-associated spectra of Kβ. The data have been smoothed with a five-point Gaussian window. b) Time-domain fits of the Kβ kinetic data from the global analysis. These have been normalised with respect to the ground state spectrum. The error bars describe the standard error of the mean (66 % confidence interval).

*Modelling $Mn_3$ TR-XES with a sequential global fit.*

Figure S8 displays the fitting results of a sequential global model to the $Mn_3$ dataset. The fit to Kα yielded two time constants of 160 fs (95 % CI 0.05 – 50 ps) and a second time constant of 9000 fs, which was fixed. Similar to the parallel model presented in the main paper, the Kβ dataset required an additional component. The fitting yielded two time constants with the value 180 fs (95 % CI 0.007 – 50 ps and 0.1 – 50 ps) and a third time constant that was fixed at 9000 fs. Given the much larger confidence intervals and that the decay associated spectra do not provide evidence of a redshift in the long-lived component seen in Figure 4 of the main text, we argue the parallel model presented in the main text is a better representation of the data.

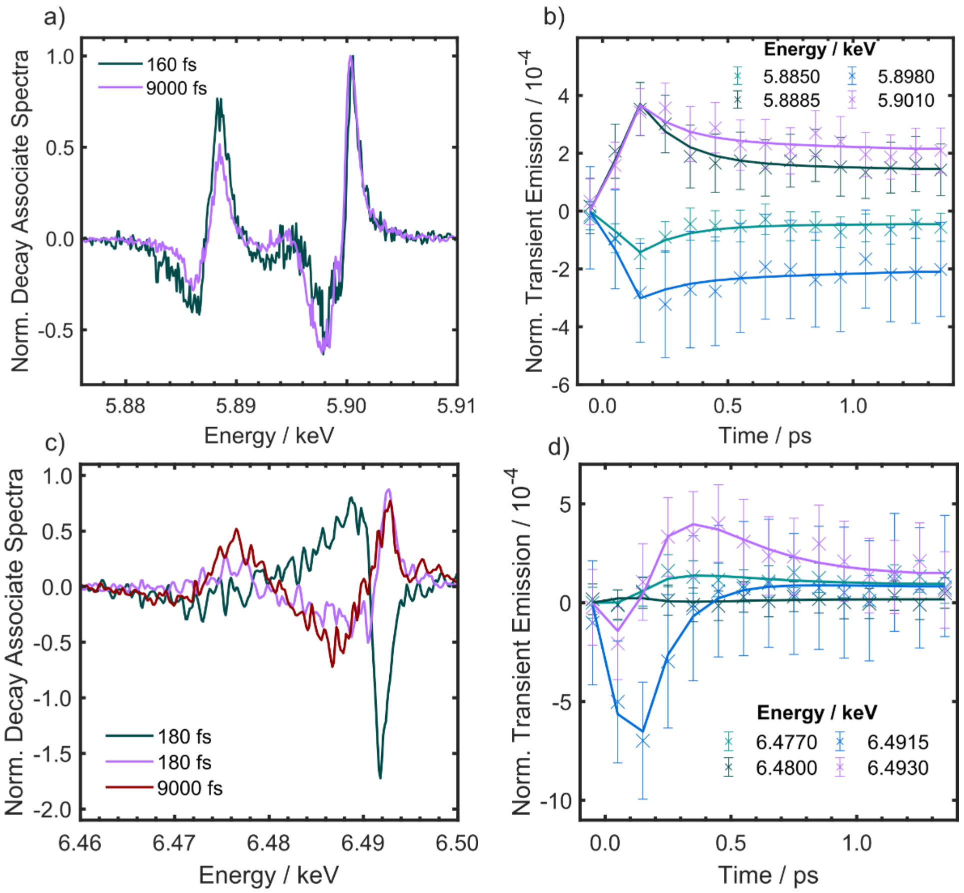


**Figure S8.** Global analysis of the TR-XES of $Mn_3$ using a consecutive model. a) Decay-associated spectra of Kα. b) Time-domain fits of the Kα kinetic data from the global analysis. These have been normalised with respect to the ground state spectrum. c) Decay-associated spectra of Kβ. The data have been smoothed with a five-point Gaussian window. d) Time-domain fits of the Kβ kinetic data from the global analysis. The error bars describe the standard error of the mean (66 % confidence interval).

**Supplementary References**


(1) Inglis, R.; Taylor, S. M.; Jones, L. F.; Papaefstathiou, G. S.; Perlepes, S. P.; Datta, S.; Hill, S.; Wernsdorfer, W.; Brechin, E. K. Twisting, Bending, Stretching: Strategies for Making Ferromagnetic [$Mn^{III}_3$] Triangles. *Dalt. Trans.* **2009**, *42*, 9157–9168.

(2) Beckwith, M. A.; Roemelt, M.; Collomb, M.-N.; DuBoc, C.; Weng, T.-C.; Bergmann, U.; Glatzel, P.; Neese, F.; DeBeer, S. Manganese Kβ X-Ray Emission Spectroscopy As a Probe of Metal–Ligand Interactions. *Inorg. Chem.* **2011**, *50* (17), 8397–8409.

(3) Lafuerza, S.; Carlantuono, A.; Retegan, M.; Glatzel, P. Chemical Sensitivity of Kβ and Kα X-Ray Emission from a Systematic Investigation of Iron Compounds. *Inorg. Chem.* **2020**, *59* (17), 12518–12535.

(4) Liedy, F.; Eng, J.; McNab, R.; Inglis, R.; Penfold, T. J.; Brechin, E. K.; Johansson, J. O. Vibrational Coherences in Manganese Single-Molecule Magnets after Ultrafast Photoexcitation. *Nat. Chem.* **2020**, *12* (5), 452–458.

(5) Biednov, M.; Yousef, H.; Otte, F.; Choi, T.-K.; Jiang, Y.; Frankenberger, P.; Knoll, M.; Zalden, P.; Ramilli, M.; Gawelda, W.; Canton, S. E.; Lima, F. A.; Milne, C.; Khakhulin, D. Hard X-Ray Emission Spectroscopy in Liquids Using MHz XFEL Source and JUNGFRAU Detectors. *Nuclear Instruments and Methods in Physics Research Section A: Accelerators, Spectrometers, Detectors and Associated Equipment* **2023**, *1055*, 168540.

(6) Neese, F. The ORCA Program System. *WIREs Comput. Mol. Sci.* **2012**, *2* (1), 73–78. https://doi.org/10.1002/wcms.81.

(7) Neese, F. Software Update: The ORCA Program System, Version 4.0. *WIREs Comput. Mol. Sci.* **2018**, *8* (1), 4–9.

(8) Adamo, C.; Barone, V. Toward Reliable Density Functional Methods without Adjustable Parameters: The PBE0 Model. *J. Chem. Phys.* **1999**, *110* (13), 6158–6170.

(9) Weigend, F.; Ahlrichs, R. Balanced Basis Sets of Split Valence, Triple Zeta Valence and Quadruple Zeta Valence Quality for H to Rn: Design and Assessment of Accuracy. *Phys. Chem. Chem. Phys.* **2005**, *7* (18), 3297.

(10) Jansen, G.; Hess, B. A. Revision of the Douglas-Kroll Transformation. *Phys. Rev. A* **1989**, *39* (11), 6016–6017.

(11) Hess, B. A. Relativistic Electronic-Structure Calculations Employing a Two-Component No-Pair Formalism with External-Field Projection Operators. *Phys. Rev. A* **1986**, *33* (6), 3742–3748.

(12) Hess, B. A. Applicability of the No-Pair Equation with Free-Particle Projection Operators to Atomic and Molecular Structure Calculations. *Phys. Rev. A* **1985**, *32* (2), 756–763.

(13) Douglas, M.; Kroll, N. M. Quantum Electrodynamical Corrections to the Fine Structure of Helium. *Ann. Phys.* **1974**, *82* (1), 89–155.

(14) Khakhulin, D.; Otte, F.; Biednov, M.; Bömer, C.; Choi, T.-K.; Diez, M.; Galler, A.; Jiang, Y.; Kubicek, K.; Lima, F. A.; Rodriguez-Fernandez, A.; Zalden, P.; Gawelda, W.; Bressler, C. Ultrafast X-Ray Photochemistry at European XFEL: Capabilities of the Femtosecond X-Ray Experiments (FXE) Instrument. *App. Sci.* **2020**, *10* (3), 995.
