# Supplementary material for: Capturing Ultrafast Spin Dynamics in Single-Molecule Magnets Using Femtosecond X-ray Emission Spectroscopy
Source: J Phys Chem Lett. 2025 Apr 17;16(17):4148–54. doi: 10.1021/acs.jpclett.5c00383 (PMC12051188; doi:10.1021/acs.jpclett.5c00383)
Supplement: Supplementary file 2 — jz5c00383_si_002.pdf [file jz5c00383_si_002.pdf]

jz-2025-00383d.R1

Name: Peer Review Information for "Capturing Ultrafast Spin Dynamics in Single-Molecule Magnets using Femtosecond X-ray Emission Spectroscopy"

First Round of Reviewer Comments

Reviewer: 1

Comments to the Author

This paper by Kyle Barlow et al reports on a study of the ultrafast dynamics of photoexcited single-molecule magnet. The authors used time-resolved K-edge X-ray emission spectroscopy (TR-XES), on a X-Ray free electron laser, to study manganese(III)-based trinuclear SMM: a first compound refered to as Mn3 and a second one, which is the model system Mn(acac)3. The authors also used complimentary NEVPT2 calculations to support their observations and interpretations.

1. What is the major advance reported in the paper?

The major advance reported in the paper is about the ultrafast photoinduced dynamics, monitored through the TR-XES spectra. The data on Mn(acac)3 are consistent with a switching between Jahn-Teller distorted structures expected after photoexcitation and they also discuss the dynamics for Mn3. The comparison of the Kβ signal reveals insight into the distribution of spin states populated within 100 fs. An important result is the attribution of different timescales associated with the photoinduced dynamics, and especially the 1100 fs time constant corresponding to a change from the excited state geometry and internal conversion back to the ground state and the 9000 fs component, which is assigned to vibrational cooling in the ground state. The results shown represent important advances, compared to previous studies on this ultrafast dynamics, which evidenced transient electronic and structural states, but which were not providing any information regarding the spin dynamics.

2. What is the immediate significance of this advance?

Achieving ultrafast photomagnetic switching of single-molecule magnets (SMMs) could lead to simultaneous fast and dense data storage devices. To facilitate this, a thorough understanding of the ultrafast dynamics emerging after ultrashort laser pulse excitation is essential. The present paper represents therefore a great topic of interest, as it opens the possibility to control anisotropy by light and therefore to induce a switch in the magnetisation direction. This topic is also important in chemistry for achieving ultrafast photomagnetic switching of single-molecule magnets materials.

3. Technical suggestions

For the general reader it would be nice to draw schematic representations of the spin configurations of the different states involved in the process (Figure 5), for the Mn3 S=6 with 3 Mn(III) ions with s = 2 ferromagnetically coupled and one or different possible excited state. It would be nice to include also in the figure the 50-80 fs spin dynamics.

Reviewer: 2

Comments to the Author

This manuscript provides an interesting study on the excited state properties of Mn complex systems that have applications as molecular magnets. While the dynamics of these complexes have been extensively studied by other optical and X-ray techniques, to my knowledge, this is the first X-ray emission spectroscopy report on such systems. This is a main focus and motivation of the paper since XES reports directly on the time resolved evolution of the spin states. It's my opinion that the system chosen and the technique are very interesting to a wide audience. However, I have some major issues with the conceptual design of the reported experiment and with some of the extrapolated conclusions. If these concerns can be addressed satisfactorily, I believe that the paper would warrant publication at the Journal of Physical Chemistry Letters.

It is my understanding that a major pillar of this study is the comparison between the excited state dynamic of the "model system" Mn(acac)3 and the SMM Mn3. This is

important since they have been observed to have vastly different excited state lifetimes due to to the axially compressed distortion of Mn(acac) that is not possible in the Mn3. However, due to the difficulty in simulating the excited states of Mn3, Mn(acac) with a artificially constrained distortion is used as a "benchmark" for the experimentally observed signals. Naively, I would not think this is a good approach. There are significant structural differences between the two complexes, and a lot of other phenomena can lead to their differences in the transient XES spectra.

Additionally, since it was clearly important to use Mn(acac) to benchmark the experimental observables and simulations, I was surprised with the choice of keeping the same excitation wavelength for both compounds, despite the claim that "the absorption cross section at the pump wavelength for Mn(acac)3 being 100 times lower than the absorption cross section at 400 nm for Mn3.". The extremely poor S/N of the Mn(acac) transient spectra is attributed to this fact, which could be easily tuned experimentally.

With that in mind, my main concern are the conclusions directly drawn form the comparison between the dynamics of Mn3 and Mn(acac). I would suggest reframing the manuscript to focus specifically on Mn3, and softening the highlighted differences relative to Mn(acac) since they are mostly within the noise level, in my opinion. I am not convinced the difference spectra are qualitatively different between the two species.

With that in mind, I still find that the conclusions from the analysis of the Mn3 are interesting, and especially the global fitting results are important to support the conclusions made by the authors.

A few specific points I would like to bring up:

- Figure 1a. Please add an inset zooming in the difference between the two lines, as in Fig1b.

- Figure 1, overall: please use a different color scheme. Blue and purple are hard to pick apart, especially given the small differences

- Add a UV-Vis spectra of both complexes to the SI, and cite them on page 6, line 125. This is also important to visualize the experimental design choices of excitation wavelength.

- Still on page 6, "The Jahn-Teller distortion initiated by the photoexcitation increases the equatorial metal-ligand bonds (1.93 to 2.07 Å), which transfers electron density towards the metal ions and decrease their effective nuclear charge and shifts the emission to higher energy." How was the bond length determined exactly?

- Is the 80 fs timescale obtained from the global analysis meaningful given the ~120 fs IRF obtained from the Fe(bpy) reference measurement?

- Finally, is it possible to provide an estimate of the population of mixed spin states during the vibrational cooling in the ground state? XES is not inherently sensitive to GS vibrational cooling, and the important assignment of the 9 ps timescale to the cooling of a hot ground state is highly dependent on the assumption that a mix of excited state spin configurations is accessible within this cooling process.

Author's Response to Peer Review Comments:

Reviewer: 1

Recommendation: This paper is publishable subject to minor revisions noted. Further review is not needed.

Comments:
This paper by Kyle Barlow et al reports on a study of the ultrafast dynamics of photoexcited singlemolecule magnet. The authors used time-resolved K-edge X-ray emission spectroscopy (TR-XES), on a X-Ray free electron laser, to study manganese(III)-based trinuclear SMM: a first compound refered to as Mn3 and a second one, which is the model system Mn(acac)3. The authors also used complimentary NEVPT2 calculations to support their observations and interpretations.

1. What is the major advance reported in the paper?
The major advance reported in the paper is about the ultrafast photoinduced dynamics, monitored through the TR-XES spectra. The data on Mn(acac)3 are consistent with a switching between JahnTeller distorted structures expected after photoexcitation and they also discuss the dynamics for Mn3.
The comparison of the Kβ signal reveals insight into the distribution of spin states populated within 100 fs. An important result is the attribution of different timescales associated with the photoinduced dynamics, and especially the 1100 fs time constant corresponding to a change from the excited state geometry and internal conversion back to the ground state and the 9000 fs component, which is assigned to vibrational cooling in the ground state. The results shown represent important advances, compared to previous studies on this ultrafast dynamics, which evidenced transient electronic and structural states, but which were not providing any information regarding the spin dynamics.

2. What is the immediate significance of this advance?
Achieving ultrafast photomagnetic switching of single-molecule magnets (SMMs) could lead to simultaneous fast and dense data storage devices. To facilitate this, a thorough understanding of the ultrafast dynamics emerging after ultrashort laser pulse excitation is essential. The present paper represents therefore a great topic of interest, as it opens the possibility to control anisotropy by light and therefore to induce a switch in the magnetisation direction. This topic is also important in chemistry for achieving ultrafast photomagnetic switching of single-molecule magnets materials.

3. Technical suggestions

For the general reader it would be nice to draw schematic representations of the spin configurations of the different states involved in the process (Figure 5), for the Mn3 S=6 with 3 Mn(III) ions with s = 2 ferromagnetically coupled and one or different possible excited state. It would be nice to include also in the figure the 50-80 fs spin dynamics.

ANSWER: We thank the reviewer for their kind comments. We have implemented the suggested changes in an updated Figure 5.

Reviewer: 2

Recommendation: This paper may be publishable, but major revision is needed; I would like to be invited to review any future revision.

Comments:
This manuscript provides an interesting study on the excited state properties of Mn complex systems that have applications as molecular magnets. While the dynamics of these complexes have been extensively studied by other optical and X-ray techniques, to my knowledge, this is the first X-ray emission spectroscopy report on such systems. This is a main focus and motivation of the paper since XES reports directly on the time resolved evolution of the spin states. It's my opinion that the system chosen and the technique are very interesting to a wide audience. However, I have some major issues with the conceptual design of the reported experiment and with some of the extrapolated conclusions. If these concerns can be addressed satisfactorily, I believe that the paper would warrant publication at the Journal of Physical Chemistry Letters.

ANSWER: We thank the reviewer for their comments. We have addressed the reviewer's comments on a point-by-point basis.

It is my understanding that a major pillar of this study is the comparison between the excited state dynamic of the "model system" Mn(acac)3 and the SMM Mn3. This is important since they have been observed to have vastly different excited state lifetimes due to to the axially compressed distortion of Mn(acac) that is not possible in the Mn3. However, due to the difficulty in simulating the excited states of Mn3, Mn(acac) with a artificially constrained distortion is used as a "benchmark" for the experimentally observed signals. Naively, I would not think this is a good approach. There are significant structural differences between the two complexes, and a lot of other phenomena can lead to their differences in the transient XES spectra.

ANSWER: The X-ray emission spectra (XES) studied here involve core-to-core transitions: 2*p*-1*s* for Kα and 3p-1*s* for Kβ. Several factors can influence the spectral shape. While we acknowledge that simulating the excited states of Mn_3 using $Mn(acac)_3$ with a constrained distortion is an approximation, we believe it is sufficient for the present analysis. However, we note that the constrained distortion is not artificial. Indeed, the distortion is consistent with the of Mn3 identified in a previous XAS study (See *Nat. Comm.* **15**:4043 [2024], ref. 34), this is not dissimilar to the Jahn-Teller distortion obverted in $Mn(acac)_3$, but due to the ligand structure in Mn_3, its magnitude is significantly reduced.

The most apparent difference between the two complexes is the presence of one Mn in $Mn(acac)_3$ versus three in Mn_3. However, the Mn atoms—and particularly their core orbitals—behave largely independently.

This is evident from the similarity in the ground-state Kα and Kβ spectra of the two complexes. As discussed in the paper, the primary difference is a slightly larger splitting between the Kβ peaks and a more intense Kβ' peak for $Mn(acac)_3$ compared to Mn_3. Each Mn(III) ion formally has a spin of *s*=2. The slightly lower intensity of the $K\beta_{1,3}$ peak in Mn_3 suggests a lower spin density on the metal ions relative to $Mn(acac)_3$.

For the transient spectra, the Kα spectra exhibit similar trends in both complexes, and this behaviour is consistently reproduced across all simulations. Since the Kα transition (2*p*-1*s*) is a deep core transition, it is well established that variations in the effective nuclear charge predominantly drive spectral changes. For Kβ, although the transitions remain core-to-core, the increased overlap between the 3*p* and 3*d* orbitals enhances the influence of exchange interactions and spin-orbit effects, as demonstrated by the ground-state spectra. Consequently, the spectral changes in Kβ are primarily governed by three key factors: (1) the effective nuclear charge on Mn, (2) the exchange interaction between the 3*p* and 3*d* electrons, and (3) the spin density of Mn.

The experimental data, our $Mn(acac)_3$ simulations, and high-level quantum chemistry simulations of the excited states presented in the paper strongly support our interpretation. While we acknowledge the approximations in our approach, our conclusions align with previous literature and are corroborated by all simulations in this work.

Additionally, since it was clearly important to use Mn(acac) to benchmark the experimental observables and simulations, I was surprised with the choice of keeping the same excitation wavelength for both compounds, despite the claim that "the absorption cross section at the pump wavelength for Mn(acac)3 being 100 times lower than the absorption cross section at 400 nm for Mn3.". The extremely poor S/N of the Mn(acac) transient spectra is attributed to this fact, which could be easily tuned experimentally.

ANSWER: Regrettably, the “100 times” lower absorption coefficient at 400 nm written in the manuscript is a mistake, the absorption is only around 10 times lower. This has been corrected in the manuscript. The optical absorption spectra have been added in the new supplementary figure (S2).

To answer the reviewer’s question: The pump wavelength was kept constant across both samples because the 400 nm excitation corresponds to the same metal-centred transition in both complexes (See. *Nat. Chem.* **12**:452–458 [2020], ref. 30). Indeed, it would be possible to excite $Mn(acac)_3$ at a wavelength where the absorption coefficient is higher. However, this corresponds to charge-transfer transitions between the metal and the ligands. We are interested in the metal-centred excitation for the purpose of changing the Jahn-Teller distortion and consequently the magnetic anisotropy. Therefore, we chose to keep the pump wavelength the same in order to compare the two complexes more directly.

With that in mind, my main concern are the conclusions directly drawn form the comparison between the dynamics of Mn3 and Mn(acac). I would suggest reframing the manuscript to focus specifically on Mn3, and softening the highlighted differences relative to Mn(acac) since they are mostly within the noise level, in my opinion. I am not convinced the difference spectra are qualitatively different between the two species.

ANSWER: The main conclusions drawn from the comparison to $Mn(acac)_3$ are:

1. **The blueshift of the transient Kα spectra are described by a change in the Jahn-Teller distortion after metal-centred excitation**. The experimental transient spectra for both complexes (Fig. 2a and 2c) and simulated data (Fig. 3c) agree well with all spectra showing a blueshift. We know from our previous work that Jahn-Teller switching occurs in Mn(acac)$_3$ (See. *Nat. Chem.* **12**:452–458 [2020], ref. 30). In this case we claim the similarity, not the differences, in the Kα spectra between the two complexes support this conclusion.
2. **The transient Kβ spectra at times 300 – 1000 fs after photoexcitation are dominated by changes induced by structural dynamics.** In this case, we argue that the data pertaining to Mn(acac)$_3$ does show a redshift (Fig. 2b) at these times, whereas Mn$_3$ shows a blueshift (Fig. 2d). These experimental observations agree well with the simulated data for both the complexes (Fig. 3d), which solely accounts for structural changes.
3. **The lack of large changes in the Kβ signal is a result of the dynamics predominately occurring within the same spin quintet manifold in Mn(acac)$_3$. The more complex spectral dynamics in the Mn$_3$ dataset are due to the involvement of many different spin states.** We agree with the reviewer that in the Mn(acac)$_3$ dataset, the more complicated spectral dynamics could be there, but just not resolved due to the lower signal-to-noise ratio compared to the Mn$_3$ dataset.

Given these points, we have made changes to the manuscript focussing mostly on point 3 as follows: On line 174:

"In contrast, the Kβ transient of Mn$_3$ exhibits much higher signal-to-noise and more complex spectral dynamics."

Has been replaced with

"The Kβ transient of Mn$_3$ exhibits much higher signal-to-noise ratio, which allows us to extract more information on the spin dynamics."

Starting on line 289 in the conclusion:

"Additionally, lack of multiple exchange-coupled metal centres means that individual spin states are well separated from one another in Mn(acac)$_3$ and retain a purer spin character. Given the spin purity, and that both the ground and lowest excited states are of quintet character, very small changes are observed in the Kβ spectra after photoexcitation that can be explained by structural changes."

Has been replaced by

"The Kβ spectra exhibits a weaker signal but the data between 300 – 700 fs can also be modelled by considering the same change in Jahn-Teller distortion which describes the Kα spectrum."

With that in mind, I still find that the conclusions from the analysis of the Mn3 are interesting, and especially the global fitting results are important to support the conclusions made by the authors.

ANSWER: We appreciate the reviewer's interest in the analysis of the Mn$_3$ data and stress that the main conclusions of the paper do relate to this molecule and that we believe comparisons to Mn(acac)$_3$ strengthen those conclusions.

A few specific points I would like to bring up:

- Figure 1a. Please add an inset zooming in the difference between the two lines, as in Fig1b.- Figure 1, overall: please use a different color scheme. Blue and purple are hard to pick apart, especially given the small differences

ANSWER: The figure has been updated with the inset and a change of colour to red/blue for easier differentiation of the lines.

- Add a UV-Vis spectra of both complexes to the SI, and cite them on page 6, line 125. This is also important to visualize the experimental design choices of excitation wavelength.

ANSWER: The optical absorption spectra of both complexes have been added to the SI in Figure SI2 and referenced in the main text.

- Still on page 6, "The Jahn-Teller distortion initiated by the photoexcitation increases the equatorial metal-ligand bonds (1.93 to 2.07 Å), which transfers electron density towards the metal ions and decrease their effective nuclear charge and shifts the emission to higher energy." How was the bond length determined exactly?

ANSWER: For $Mn(acac)_3$, there is no experimental excited-state structural data available and the bond length changes are calculated at CASSCF//NEVPT2 level by performing geometry optimisations in the ground and first excited states (See *Nat. Chem.* **12**:452–458 [2020], ref. 30). For bond lengths relating to Mn_3, the bond lengths have been experimentally determined (See *Nat. Comm.* **15**:4043 [2024], ref. 34). To clarify this point we have replaced the following text:

“The Jahn-Teller distortion initiated by the photoexcitation increases the equatorial metal-ligand bonds (1.93 to 2.07 Å), which transfers electron density towards the metal ions and decrease their effective nuclear charge and shifts the emission to higher energy.” with

“The Jahn-Teller distortion initiated by the photoexcitation increases the equatorial metal-ligand bonds from 1.93 to 2.07 Å as shown by CASSCF//NEVPT2 calculations.[30] This transfers electron density towards the metal ions and decrease their effective nuclear charge and shifts the emission to higher energy.”

- Is the 80 fs timescale obtained from the global analysis meaningful given the ~120 fs IRF obtained from the Fe(bpy) reference measurement?

ANSWER: We thank the reviewer for pointing this out. The exact value of 80 fs is perhaps not physically meaningful. However, given the clear difference between the 50 fs and 200 fs spectra in Figure 2d, there is certainly a distinct dynamic process occurring on these timescales. We have added the following to the manuscript on line 231:

“Additionally, given the instrument response function determined by reference measurements is 120 fs (see SI, Figure SI6), the exact value of 80 fs is not physically meaningful. However, given the difference between the spectra at 50 fs and 200 fs there is clearly a distinct process occurring at these short timescales.”

- Finally, is it possible to provide an estimate of the population of mixed spin states during the vibrational cooling in the ground state? XES is not inherently sensitive to GS vibrational cooling, and the important assignment of the 9 ps timescale to the cooling of a hot ground state is highly dependent on the assumption that a mix of excited state spin configurations is accessible within this cooling process.

ANSWER: In these exchanged-coupled complexes, the spin states are very closely spaced, smaller to the spacing of vibrational levels. The energy of the first excited spin state was calculated from magnetic susceptibility measurements as 2 meV (16 cm^{-1}) (See *Dalt. Trans* **42**:9157-9168 [2009], ref. 35). Additionally, these states have been calculated at NEVPT2 level and presented in Table S2, which shows there are many closely-spaced spin states. This is why we argue we are sensitive to cooling dynamics in XES as a hot ground state will have both a distribution of populated vibrational and spin states and XES is sensitive to the spin states. We have added the following sentence on line 284 to the manuscript to clarify:

"Although XES is not inherently sensitive to vibrational cooling, in these exchange-coupled complexes the energy spacing of the spin states is lower than that of typical vibrational modes (< 100 cm^{-1}). These have been calculated and presented in Table S2. Therefore, cooling will also occur through different spin states, which XES is sensitive to."

jz-2025-00383d.R2

Name: Peer Review Information for "Capturing Ultrafast Spin Dynamics in Single-Molecule Magnets using Femtosecond X-ray Emission Spectroscopy"

Second Round of Reviewer Comments

Reviewer: 2

Comments to the Author

The changes made to the manuscript by the authors have satisfied my questions and concerns. I recommend the publication of the revised version.

Reviewer: 1

Comments to the Author

This paper by Kyle Barlow et al "Capturing Ultrafast Spin Dynamics in Single-Molecule Magnets using

Femtosecond X-ray Emission Spectroscopy" reports on a study of the ultrafast dynamics of photoexcited single-molecule magnet.

The photomagnetic effect has received a great deal of attention in condensed matter physics, especially regarding potential applications in opto- or spin-tronics. The possibility to control anisotropy by light and therefore to induce a switch in the magnetisation direction is very promising. In this work, the authors focus on single-molecule magnets (SMMs) with Jahn-Teller (JT) distortions that can be manipulated by light—and thereby alter magnetic anisotropy, which was already studied by some if the authors with optical spectroscopy.

In the present study, Barlow et al. employ time-resolved X-ray emission spectroscopy (XES) to probe spin dynamics relevant to the photoinduced changes in magnetic anisotropy mediated by the JT distortions, driven by an optical excitation with 400 nm (d-d transition), on manganese(III)-based trinuclear SMM: The so-called Mn3 systems and the so-called model system Mn(acac)3. The TR-XES spectra on Mn(acac)3 are consistent with switching between Jahn-Teller distorted structures expected after photoexcitation and they also discuss the dynamics for Mn3. The comparison of the Kβ signal reveals insight into the distribution of spin states populated within 100 fs.

This study, by bringing key X-ray based spectroscopy information about spin and charge dynamics contributes to the ongoing exploration of photomagnetic effects in SMMs. I recommend to publish the work as it is, considering the minor points mentioned below:

Minor comments for improving presentation:

1. Figure 2 and Figure 3 : I recommend to add “Mn(acac)3” and “Mn3” on each panels to make the Figures easier to read.

2. Figure 4b and d: the error bars look large compared to the apparent quality of the spectral changes. Is it a choice of the authors?.

Congratulations to the authors, this is a very nice piece of work.

Author's Response to Peer Review Comments:

Dear Prof. Zhang,

Thank you for handling our article. Below, we have responded to the two comments of reviewer 1. We have also read through the article and corrected typographical errors.

Best wishes,

Kyle Barlow, on behalf of all the authors.

Reviewer: 1

Recommendation: This paper is publishable subject to minor revisions noted. Further review is not needed.

Comments:

This paper by Kyle Barlow et al "Capturing Ultrafast Spin Dynamics in Single-Molecule Magnets using

Femtosecond X-ray Emission Spectroscopy" reports on a study of the ultrafast dynamics of photoexcited single-molecule magnet.

The photomagnetic effect has received a great deal of attention in condensed matter physics, especially regarding potential applications in opto- or spin-tronics. The possibility to control anisotropy by light and therefore to induce a switch in the magnetisation direction is very promising. In this work, the authors focus on single-molecule magnets (SMMs) with Jahn-Teller (JT) distortions that can be manipulated by light—and thereby alter magnetic anisotropy, which was already studied by some if the authors with optical spectroscopy.

In the present study, Barlow et al. employ time-resolved X-ray emission spectroscopy (XES) to probe spin dynamics relevant to the photoinduced changes in magnetic anisotropy mediated by the JT distortions, driven by an optical excitation with 400 nm (d-d transition), on manganese(III)-based trinuclear SMM: The so-called Mn3 systems and the so-called model system Mn(acac)3. The TR-XES spectra on Mn(acac)3 are consistent with switching between Jahn-Teller distorted structures expected after photoexcitation and they also discuss the dynamics for Mn3. The comparison of the Kβ signal reveals insight into the distribution of spin states populated within 100 fs.

This study, by bringing key X-ray based spectroscopy information about spin and charge dynamics contributes to the ongoing exploration of photomagnetic effects in SMMs. I recommend to publish the work as it is, considering the minor points mentioned below:

Minor comments for improving presentation:

1. Figure 2 and Figure 3 : I recommend to add "Mn(acac)3" and "Mn3" on each panels to make the Figures easier to read.

We have added these captions to Figures 2 and 3.

2. Figure 4b and d: the error bars look large compared to the apparent quality of the spectral changes. Is it a choice of the authors?

The spectral changes shown in Figure 2 were obtained at a fixed time delay, with each measurement typically averaged over a period of 30 to 60 minutes. In contrast, Figure 4 presents data from time-resolved measurements, where the time delay was scanned and each time point was averaged for only a few minutes. As a result, the standard error of the mean (used for the error bars in both figures) is smaller in Figure 2 due to the larger number of measurements contributing to each data point. These measurement times are provided in the methods found in the supplementary information.

Congratulations to the authors, this is a very nice piece of work.

We thank the reviewer for their kind comments on the manuscript.
